# Lipoprotein(a) Lipidome: Responses to Reduced Dietary Saturated Fat Intake in Two Randomized Controlled Feeding Trials

**DOI:** 10.3390/nu17193113

**Published:** 2025-09-30

**Authors:** Munkhtuya Myagmarsuren, Hayley G. Law, Wei Zhang, Tselmen Anuurad, Heejung Bang, Lauren M. Bishop, Tong Shen, Oliver Fiehn, Kristina S. Petersen, Lars Berglund, Byambaa Enkhmaa

**Affiliations:** 1 Department of Internal Medicine, University of California-Davis, Davis, CA 95616, USAwzhang@health.ucdavis.edu (W.Z.);; 2 Department of Public Health Sciences, University of California-Davis, Davis, CA 95616, USA; 3 West Coast Metabolomics Center, University of California-Davis, Davis, CA 95616, USA; 4 Department of Nutritional Sciences, The Pennsylvania State University, University Park, PA 16802, USA

**Keywords:** diet, Lp(a), oxidized phospholipids, Lp(a)-lipidomics, risk properties

## Abstract

**Background/Objectives**: An elevated level of lipoprotein(a) [Lp(a)] is a genetically determined risk factor for cardiovascular disease. The atherogenic properties of Lp(a) include attribution to its role as a carrier of oxidized phospholipids (OxPL). Despite genetic control, Lp(a) levels increase with dietary saturated fat (SFA) reduction. However, little is known about the impact of dietary factors on Lp(a) risk properties. **Methods**: We assessed total Lp(a)-OxPL concentration, Lp(a)-OxPL subspecies abundance, and Lp(a) lipidomics in response to SFA reduction in two multicenter, randomized, controlled, crossover feeding trials, DELTA (Dietary Effects on Lipoproteins and Thrombogenic Activity) 1 (96 healthy individuals) and 2 (79 metabolically challenged individuals). In both trials, significant increases in Lp(a) levels were reported previously. **Results**: While no between-diet differences in the concentrations of total Lp(a)-OxPL and four major OxPL subspecies (ALDOPC, POVPC, PAzPC, and PGPC) were observed in DELTA 1, ALDOPC decreased significantly in DELTA 2 when SFA was replaced with carbohydrates (*p* = 0.014). Of 440 individual lipid species annotated in an untargeted analysis of the Lp(a) lipidome, 87 lipids differed significantly (*p* < 0.05 adjusted for multiplicity) between diets, with triacylglycerol species showing the most pronounced changes in both trials. For all intervention diets, triacylglycerol species with a higher average number of carbon atoms and double bonds increased the most in abundance with SFA reduction. **Conclusions**: In parallel with an increase in plasma Lp(a) levels, significant changes in Lp(a) lipid composition occurred. The findings demonstrate the dynamic nature of intraindividual Lp(a) lipid composition in response to diet interventions.

## 1. Introduction

An elevated lipoprotein(a) [Lp(a)] concentration is an independent, causal, and prevalent risk factor for atherosclerotic cardiovascular disease (CVD) [1]. It is well documented that the size variability of the apolipoprotein(a) [(apo(a)] component is a critical factor in determining Lp(a) plasma concentrations and hence its atherogenic potential [2,3,4]. Mechanisms underlying the atherogenic properties of Lp(a) are associated with its role as a carrier of proinflammatory and proatherogenic oxidized phospholipids (OxPL) [5,6,7].

While Lp(a) concentration is under strong genetic regulation [4], it has become clear that some clinical and environmental factors impact Lp(a) [8,9,10,11]. Among the latter, intake of saturated fats (SFA) influences Lp(a) concentrations [12,13,14]. Evidence from several well-designed controlled dietary studies, including the DELTA (Dietary Effects on Lipoproteins and Thrombogenic Activity) 1 and 2 trials, demonstrates significant increases in Lp(a) concentration (11−20%) following SFA reduction [12,13]. These findings suggest metabolic regulation of Lp(a) concentrations. However, little is known about the influence of dietary factors on Lp(a)’s risk properties, such as OxPL and/or other lipidomic components.

To address these knowledge gaps, we conducted a comprehensive assessment of Lp(a)-OxPL concentration, Lp(a)-OxPL subspecies abundance, and Lp(a) lipidomics data in response to dietary SFA reduction. We utilized the DELTA program, which comprised two multicenter, randomized, controlled, crossover metabolic feeding trials. While DELTA 1 examined the effects of reducing SFA intake in healthy individuals using a stepwise approach [from 16% in an average American diet (AAD) to 9% and further to 5%] with a corresponding increase in complex carbohydrates [12], DELTA 2 was designed to specifically test the effects of replacing SFA with either carbohydrates or monounsaturated fats (MUFA) in metabolically at-risk individuals [13]. These unique study designs, coupled with the use of a comparable AAD in both DELTA trials, enabled the testing of similarities and heterogeneity in the intraindividual responses across the two trials. To the best of our knowledge, this is the first comprehensive study to systematically evaluate an array of Lp(a) lipidomic properties, moving beyond a simple measurement of Lp(a) concentration, to advance our understanding of the metabolic impact of a common dietary intervention recommended at the population level for CVD risk reduction.

## 2. Materials and Methods

### 2.1. Study Participants

The recruitment protocols and study designs of DELTA 1 and 2 have been reported previously [12,13]. Briefly, DELTA 1 recruited normolipidemic participants aged 22−67 years in four geographical locations (Columbia University, Pennington Biomedical Research Center, Pennsylvania State University, and University of Minnesota) [12]. In DELTA 1, mean plasma total cholesterol levels, obtained after a 12 to 14 h fasting on two occasions, had to be between the 25th and 90th percentiles for age, race, and sex [15]. Plasma fasting triglycerides and HDL-C, measured at the last screening visit, had to be below the 90th and above the 10th percentile, respectively. The DELTA 2 study recruited participants aged 21−61 years from the same four geographical locations [13]. DELTA 2 recruited participants who were at-risk based on the components of the metabolic syndrome. Thus, participants were eligible if the average of two screening measurements met any of the following requirements based on the criteria of the third National Health and Nutrition Examination Survey specific for age, sex, and race: (1) HDL-C ≤30th percentile, (2) triacylglycerol ≥70th percentile, and (3) insulin ≥70th percentile. Individuals were ineligible if their (1) average screening total cholesterol was <25th percentile or >90th percentile, (2) LDL-C was >4.91 mmol/L, (3) fasting triacylglycerol concentrations were <30th percentile or >5.65 mmol/L, or (4) HDL-C was >70th percentile [13]. In accordance with the recruitment strategy, the study cohort was characterized by having, at screening, low HDL-C, moderately elevated fasting TG and insulin, and near normal LDL-C, as reported previously [13]. In both DELTA studies, participants were required to be in good health, free of chronic diseases, including diabetes mellitus, and not taking medications known to affect lipids or thrombotic factors. Baseline lipid and lipoprotein levels for both trials have been previously reported [16]. Participants were required to comply with dietary guidelines, and the order of diet interventions was randomized for each participant. The study protocols were approved by the Institutional Review Boards at each of the study sites [12,13]. The studies abide by the Declaration of Helsinki principles. The CONSORT reporting guidelines for randomized trials were followed [17], and the participant recruitment processes in both DELTA studies are shown in Supplemental Figure S1.

### 2.2. Intervention Diets

Details of each study’s intervention diets, including nutrient intake, have been reported previously [12,13]. Diet compositions in DELTA 1 and 2 trials are shown in Supplemental Table S1. Briefly, the DELTA 1 protocol was designed to determine the effects of reducing total fat, particularly SFA, on lipids, lipoproteins, and thrombogenic factors [12]. Intervention diets included a diet similar to average American intake at the time (AAD) with 37% kcal of total fat [16% kcal SFA, 14% MUFA, and 7% polyunsaturated fats (PUFA)], a Step-1 Diet with 30% kcal of total fat [9% kcal SFA, 14% kcal MUFA, and 7% kcal PUFA], and a Low-Saturated-Fat Diet (Low-Sat) with 26% kcal of total fat [5% kcal SFA, 14% kcal MUFA, and 7% kcal PUFA]. Intake of carbohydrates was 48%, 55%, and 59% kcal for the AAD, Step-1, and Low-Sat diets, respectively. Trans-fatty acid levels at <1.5% kcal, intake of 300 mg/d of dietary cholesterol, and an intake of 15% kcal from protein were maintained across all diets. The profile of saturated fatty acids consumed by participants was previously reported [16]. Briefly, palmitic acid (C16:0) was the major SFA, with its contribution increasing from 51% on the AAD to 60% on the Step-1 diet and 64% on the Low-Sat diet in DELTA 1. Stearic acid (C18:0) increased from 18% to 22% to 26% of saturated fat calories across the three diets. Lauric (C12:0) and myristic (C14:0) acids together decreased from 24% to 13% to 6% across the three diets [16]. The DELTA 2 protocol, while similar, was designed to specifically test the effects of replacing SFA primarily with carbohydrates or MUFA [13]. A similar AAD diet was used, with 37% kcal of total fat [16% kcal SFA, 14% MUFA, and 7% PUFA]; for the diet replacing SFA primarily with MUFA (MUFA diet), the total fat was 37% kcal [8% kcal SFA, 22% kcal MUFA, and 7% kcal PUFA]; for the diet replacing SFA primarily with carbohydrates (CHO diet), total fat was 30% kcal [8% kcal SFA, 15% kcal MUFA, and 7% kcal PUFA]. Carbohydrate intake was 47%, 47%, and 54% kcal for the AAD, MUFA, and CHO, respectively. Similar to the DELTA 1 protocol, cholesterol (300 mg/d) and protein (16% kcal) contents were held constant between the interventions. In both DELTA trials, each diet period was 7 to 8 weeks long, with a 4 to 6 week washout period between diet periods. Dietary compliance was assessed by tray check observations (for meals eaten at the study sites) and self-reported questionnaire forms for packed meals. As previously reported, the diets were well accepted by the participants, and dietary compliance, as assessed by daily records and interviews, was outstanding at all sites [16]. In both trials, the intervention diets with a lower intake of SFA resulted in a significant ~7–11% reduction in LDL-C concentrations [12,13].

### 2.3. Determinations of Concentrations of Lp(a) and Oxidized Phospholipids Bound to Lp(a) in Plasma

Plasma Lp(a) concentrations in DELTA 1 and 2 were measured by enzyme-linked immunosorbent assay as described previously [12,13]. OxPLs bound to Lp(a) [Lp(a)-OxPL] were measured using an immunoassay protocol with two different antibodies, as in our previous study [18], in plasma samples collected during the final week of each intervention diet (week 8 in DELTA 1 and week 7 in DELTA 2). These samples were stored at −80 °C at all times and were not previously thawed. Briefly, the E06 anti-OxPL antibody (Absolute Antibody NA, MA, Boston, USA), which binds to non-native OxPLs via the phosphorylcholine (PC) head group [19,20,21], was incubated overnight with MagnaBind Goat Anti-Rabbit IgG (Rockford, IL, USA) magnetic beads in a 50:1 ratio, forming a complex [5]. Then, samples were analyzed using a second anti-Lp(a) antibody directed against the non-repeating region of apo(a). The results of this assay were referred to as Lp(a)-OxPL total concentration in plasma [18].

### 2.4. Lp(a)-Bound Oxidized Phospholipids Subspecies and Lp(a) Lipidomics Analyses

We isolated Lp(a) from plasma samples collected during the final week of each intervention diet by immunoprecipitation, as in our previous study [22]. The plasma samples were stored at −80 °C at all times and were not previously thawed. We determined Lp(a) values in the isolated samples and found an average recovery rate of ~76% and strong positive correlations with the plasma Lp(a) concentrations (r = 0.743−0.834 in DELTA 1; r = 0.694−0.854 in DELTA 2 across diet interventions, *p* < 0.001 for all). Isolated Lp(a) collected in methanol (100 µL) with an added pre-chilled antioxidant 0.01% (*w*/*v*) butylated hydroxytoluene (BHT) was used for further analyses. A predetermined set of four major OxPLs containing phosphocholine (OxPC), including 1-palmitoyl-2-(9′-oxo-nonanoyl)-sn-glycero-3-phosphocholine (ALDOPC), 1-palmitoyl-2-(5′-oxo-valeroyl)-sn-glycero-3-phosphocholine (POVPC), 1-palmitoyl-2-azelaoyl-sn-glycero-3-phosphocholine (PAzPC), and 1-palmitoyl-2-glutaryl-sn-glycero-3-phosphocholine (PGPC) [23] was analyzed using a Q-Exactive HF (Thermo Fisher Scientific, Bremen, Germany), high resolution liquid chromatography tandem mass spectrometry (LC-MS/MS). These OxPC subspecies were selected as they have been shown to be associated with Lp(a)/apo(a) and contribute to Lp(a) atherogenicity via arterial inflammation [23].

Fifteen µL was aliquoted from each sample to create the pool quality control (pool QC). The remaining 85 µL of sample and pool QC was dried in a centrivap. Prior to LC-MS/MS analysis, the lipid extract was resuspended in 50 µL of MeOH–Toluene (9:1, *v*/*v*) containing internal standard mix (UltimateSPLASH™ ONE, Avanti Polar Lipids (Alabaster, AL, USA) 1:250 diluted). Lipids were separated on a Waters ACQUITY Premier BEH C18 Column, 1.7 µm, 2.1 × 50 mm, by a gradient from 15% to 99% of mobile phase B over 5 min at 65 °C at a flow rate of 0.8 mL/min. Mobile phase A was 60:40 (*v*/*v*) acetonitrile–water with 10 mM ammonium formate and 0.1% formic acid; mobile phase B was 90:10 (*v*/*v*) isopropanol–acetonitrile with the same modifier. We used positive electrospray ionization mass spectrometry on a Thermo Fisher Q-Exactive HF mass spectrometer (Bremen, Germany), acquiring accurate mass in the 120–1700 *m*/*z* range at 60,000 mass resolution, using capillary voltage +3.5 kV and nitrogen gas temperature 380 °C. Tandem mass spectra were acquired by top2 data-dependent acquisition. For OxPL targets, we optimized all parallel reaction monitoring conditions using commercial standards of POVPC, PGPC, PAzPC, and ALDOPC. Raw data accurate mass MS/MS files were processed by AWS (Seattle, WA, USA) computing based LC-BinBase pipeline for peak picking, with the following parameters: retention time 0–5 min; MS1 tolerance 0.005 Da; smoothing level 3 scans; min peak height 10K amplitude; entropy similarity score > 70% [24]. Recursive data analysis was used to search and replace missing values from raw data files. Data processing by LC-BinBase enabled searching all public mass spectral libraries, including MS-DIAL, LipidBlast, NIST23, MassBank.us, Agilent, and GNPS. We obtained annotated lipids by matching accurate mass and diagnostic fragments from the tandem mass spectrum and LC retention time. Samples were injected in a randomized order. Pool QC was injected periodically to correct for technical variations. Five-point calibration curve of the four OxPCs, ranging from 1 to 100 ng/mL, was injected and used to quantify the four OxPL subspecies. We estimated the OxPC subspecies per unit Lp(a) by dividing each of the four OxPC masses in an isolated Lp(a) sample by the corresponding concentration of isolated Lp(a), expressed as grams per unit of Lp(a) (g/U) (i.e., particle concentration). The Lp(a) lipidomics data were normalized using the sum of all the annotated lipids, and fold differences were calculated for each lipid species comparing the different diets, as shown below.

### 2.5. Statistics

Standard descriptive statistics were used to summarize data with mean and standard deviation (SD) or median and interquartile range (IQR) for continuous variables and frequency and proportion for categorical variables. The median and IQR were used to describe non-normally distributed data. For data comparisons, non-normally distributed data were square-root transformed prior to analysis. We used a one-way ANOVA test for differences between three diet periods and a two-tailed, paired Student’s t-test for differences between two diet periods. We calculated the fold change (FC) in Lp(a) lipid species as follows: FC = (median Step-1 diet/median AAD or median Low-Sat diet/median AAD) in the DELTA 1 trial, and FC = (median MUFA/median AAD or median CHO/median AAD) in the DELTA 2 trial. The fold changes were transformed using log_2_ transformation to simplify data interpretation [25], as positive normalized log_2_ transformed FC (log_2_FC) values indicate an increase, negative log_2_FC values indicate a decrease, and log_2_FC values equal to zero indicate no change in abundance between the two diet periods. The fold changes were visualized in a heatmap, highlighting the lipid species with significant changes between the AAD (the reference diet) and either the Step-1 diet or the Low-Sat diet in DELTA 1 and between the AAD and the MUFA diet or the CHO diet in DELTA 2. All *p*-values from the lipidomics data analyses were adjusted using Benjamini and Hochberg false discovery rate correction [26]. An adjusted *p*-value less than 0.05 was considered statistically significant. Principal component analysis (PCA) was calculated using all annotated lipids from a cube root-transformed, auto-scaled dataset. PCA plots were generated using MetaboAnalyst 6.0. We also performed exploratory analyses using a mixed-effect model to assess effects of diet, diet period, diet sequence, and diet x period interaction in each DELTA study. In these analyses, we included eight commonly changed lipids across all intervention diets in both DELTA trials in addition to the Lp(a) level, Lp(a)-OxPL total concentration, and sum of the four major OxPC species. Finally, we performed Pearson’s correlation test between changes in Lp(a) levels and measures of Lp(a)-OxPL (absolute changes) and most significantly increased or decreased lipid species (log_2_FC) in response to intervention diets in each trial. SAS version 9.4 was used for analyses (SAS Institute, Cary, NC, USA).

## 3. Results

### 3.1. Participant Characteristics

Participant characteristics and changes in Lp(a) concentrations in the original DELTA 1 (n = 103) and DELTA 2 (n = 85) cohorts have been reported previously [12,13]. The present report is based on data from participants with available samples for lipidomic analyses, including 96 DELTA 1 and 79 DELTA 2 participants (Supplemental Figure S1). Briefly, the DELTA 1 cohort had a mean age of 38 ± 14 years, 54% females, 26% Black participants, and an average BMI of 24.5 ± 3.2 kg/m^2^ (Supplemental Table S2). The median (IQR) Lp(a) concentration at the end of the AAD was 11 (5; 30) mg/dL. The average increase in Lp(a) between the AAD and Step-1 diet was 1.0 ± 4.0 mg/dL (corresponding to a relative increase of 15% ± 26%), and the corresponding increase between the AAD and Low-Sat diet was 3.0 ± 6.0 mg/dL (relative increase: 24% ± 35%). The DELTA 2 study cohort had 10% Black participants and 34% females, with a mean age of 35 ± 9 years and BMI of 27.7 ± 4.5 kg/m^2^ (Supplemental Table S2). The median (IQR) Lp(a) concentration at the end of the AAD was 7 (2; 17) mg/dL. In this cohort, the median Lp(a) concentrations were higher during both the MUFA and CHO diets compared with the AAD, with a relative corresponding increase in (mean ± SD) 26% ± 63% and 19% ± 41%, respectively.

### 3.2. Responses of Total Concentration and Individual Subspecies’ Abundance of Lp(a)-Bound Oxidized Phospholipids to Dietary Saturated Fat Reduction

In DELTA 1, Lp(a)-OxPL total concentrations [median (IQR)] were 8.3 (5.7; 10.5) U/L, 7.5 (5.3; 10.3) U/L, and 8.0 (5.4; 9.7) U/L for the AAD, Step-1 diet, and Low-Sat diet interventions, respectively, without significant differences between interventions (Table 1). In DELTA 2, Lp(a)-OxPL total concentrations were 10.1 (7.31; 12.6) U/L at the end of the AAD, 7.7 (6.1; 12.2) U/L at the end of the MUFA diet, and 10.0 (7.0; 12.1) U/L at the end of the CHO diet (*p* = 0.413 for between-diet differences) (Table 1).

In both DELTA trials, the most abundant Lp(a)-OxPL subspecies was ALDOPC, followed by, in descending order of abundance, POVPC, PAzPC, and PGPC (Table 1). In DELTA 1, the sum of the four Lp(a)-OxPL subspecies was not significantly different when comparing Step-1 and Low-Sat diets with AAD (*p* > 0.05 for all). Similarly, there were no significant differences in concentrations between the diets for individual OxPL subspecies, including ALDOPC and POVPC for Step-1 and Low-Sat diets, respectively, when compared to the AAD. In DELTA 2, neither the sum of four Lp(a)-OxPL subspecies nor any of the individual subspecies differed significantly between the AAD and the MUFA diet (*p* > 0.05 for all). However, the concentrations of ALDOPC (*p* = 0.014) and the sum of the four major Lp(a)-OxPL subspecies (*p* = 0.028) decreased significantly when comparing the CHO diet with AAD (Table 1).

### 3.3. Lp(a) Lipidomics Response to Dietary Saturated Fat Reduction

**Figure 1:**
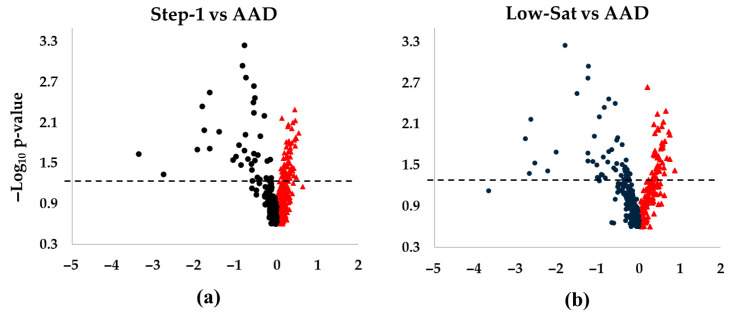

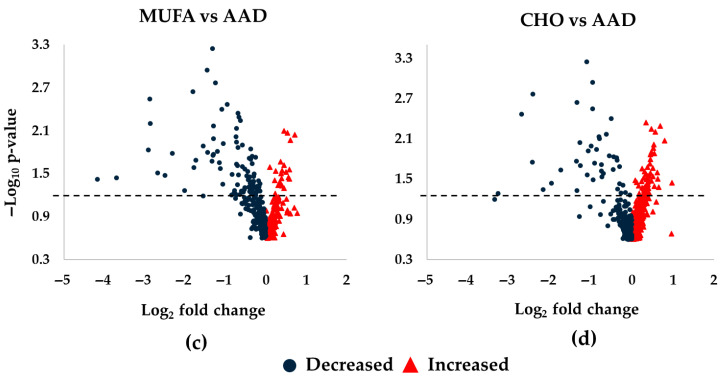
Differences in Lp(a) lipid changes following diet interventions and statistical significance in DELTA 1 and 2. The volcano plots show increased and decreased individual lipid species in Lp(a) following the respective intervention diets compared to the average American diet (AAD) in the DELTA studies. (**a**) Step-1 diet compared to AAD in DELTA 1; (**b**) Low-Sat diet compared to AAD in DELTA 1; (**c**) MUFA diet compared to AAD in DELTA 2; (**d**) CHO diet compared to AAD in DELTA 2. The dashed lines mark *p* = 0.05, with points above the line having *p* < 0.05 and points below the line having *p* > 0.05. One non-significant lipid species in panel (**b**) was not shown due to scaling (Log_2_FC of ~5.5). False discovery rate corrected *p*-values were –log_10_ transformed. Abbreviations: CHO, carbohydrate; MUFA, monounsaturated fatty acids.

An overview of the Lp(a) lipidomics response to SFA reduction by intervention diets in DELTA 1 and 2 is shown as volcano plots in Figure 1. Overall, our Lp(a) lipidomics analysis annotated 440 unique lipid species across 20 lipid classes. Among them, 87 species (20%) showed significant changes in their abundance in response to intervention diets compared with the AAD in both DELTA studies. Notably, there was a consistent pattern in the Lp(a) lipidome response across all diet interventions, with greater degrees of changes and significance observed for the decreased vs. increased lipid species (Figure 1a−d). The proportion of significantly altered lipid species (total, increased, or decreased) for the eight largest lipid classes is shown in Supplemental Table S3. Below, we describe detailed Lp(a) lipidomics findings in each of the DELTA studies.

DELTA 1: The major lipid classes represented triacylglycerol (TG) (n = 90 different species), sphingomyelin (SM) (n = 83), phosphatidylcholine (PC) (n = 74), and alkylphosphatidylcholine (PC-O) (n = 73). Due to method optimization restrictions, cholesteryl esters and cholesterol were not considered in our analyses. In terms of relative abundance, PC dominated, followed by TG, SM, and lysophosphatidylcholine (LPC) in descending order. This pattern remained consistent across all three diets, with the highest overall relative lipid abundance observed for the Step-1 diet. While more lipid species increased (n = 56) vs. decreased (n = 31) during the Step-1 diet vs. the AAD, the number of increased (n = 42) vs. decreased (n = 45) lipids was similar during the Low-Sat diet vs. AAD (Figure 1a,b). The major lipid classes that contributed to the observed significant changes during both intervention diets were TG (>40 species) and PC (>18 species) lipid classes.

**Figure 2:**
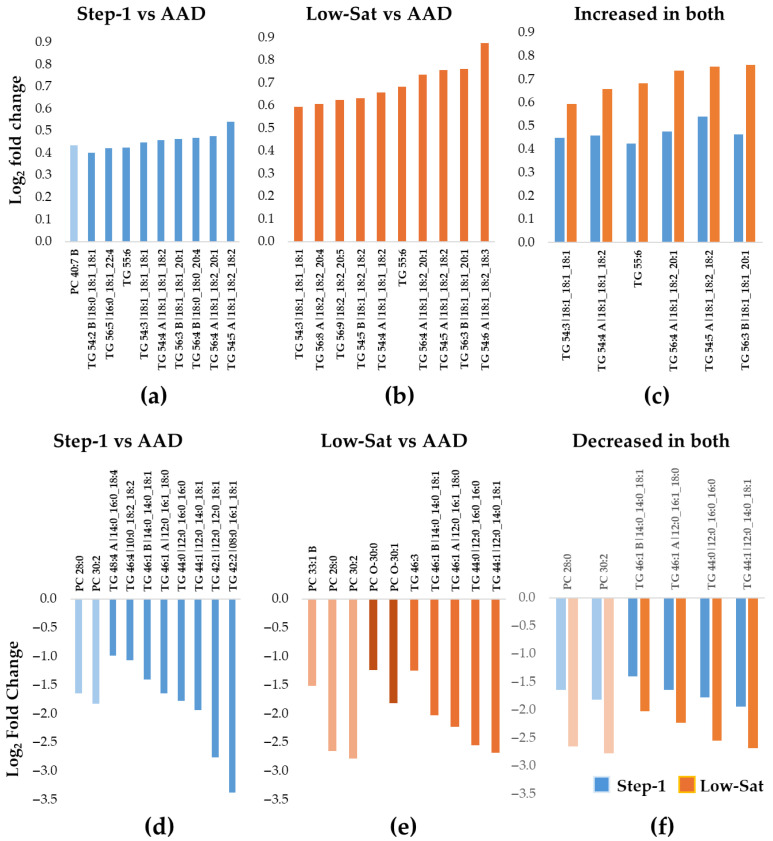
Ten most increased or decreased Lp(a) lipids during the Step-1 and the Low-Sat diets compared with the average American diet in DELTA 1. The bar graphs show the median fold increase (**a**–**c**) or decrease (**d**–**f**) for the 10 most significantly changed lipids relative to the average American diet (AAD) (adjusted *p* < 0.05 for all). (**a**) Step-1 diet compared to AAD; (**b**) Low-Sat diet compared to AAD; (**c**) among the 10 most increased lipids, 6 TGs increased during both diet interventions, with a larger increase in the Low-Sat diet vs. the Step-1 diet. The blue color represents data for Step-1 vs. AAD and the orange color represents data for Low-Sat vs AAD. (**d**) Step-1 diet compared to AAD; (**e**) Low-Sat diet compared to AAD; (**f**) among the 10 most decreased lipids, a total of 6 lipids (2 PCs and 4 TGs) decreased during both diet interventions, with a greater decrease in the Low-Sat diet vs. the Step-1 diet. The blue color represents data for Step-1 vs AAD and the orange color represents data for Low-Sat vs AAD Abbreviations: Lp(a), lipoprotein(a); PC, phosphatidylcholine; TG, triacylglycerol; Sat, saturated.

The 10 most significantly increased lipid species during the Step-1 diet included 9 longer-chain fatty acid TGs and 1 PC (Table 2) (Figure 2a), while all 10 lipid species that changed the most in response to the Low-Sat diet were TGs (Table 3) (Figure 2b). Six TGs increased during both interventions compared to AAD, with an overall greater increase for the Low-Sat diet (Table 2) (Figure 2c). In contrast, the pattern for the 10 lipids that decreased most in abundance was dominated by shorter-chain TGs as well as PCs (Table 2). Specifically, eight TGs and two PCs (Figure 2d) and five TGs, three PCs, and two PC-Os (Figure 2e) were among the lipids that decreased the most in abundance following the Step-1 diet (Table 2) and the Low-Sat diet (Table 3), respectively. Of the 10 lipids that decreased most in abundance during both intervention diets compared with the AAD, 6 were seen during both diet interventions (4 shorter-chain TGs and 2 PC species) (Figure 2f).

**Figure 3:**
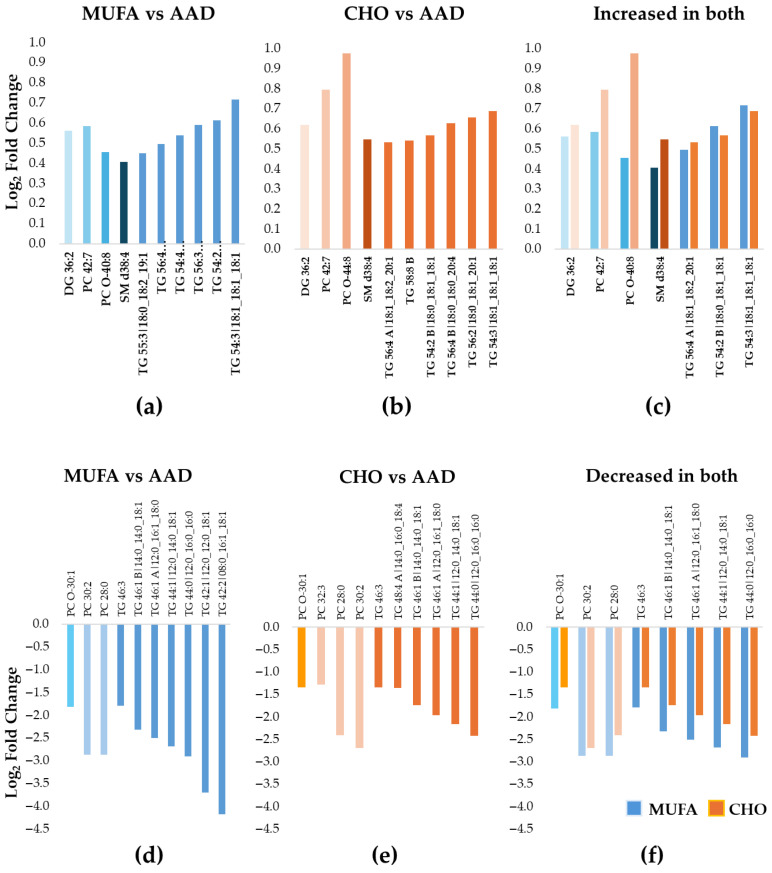
Ten most increased or decreased Lp(a) lipids during the MUFA and the CHO diets compared with the average American diet in DELTA 2. The bar graphs show the median fold increase (**a**–**c**) or decrease (**d**–**f**) for the 10 most significantly changed lipids relative to the average American diet (AAD) (adjusted *p* < 0.05 for all). (**a**) MUFA diet compared to AAD; (**b**) CHO diet compared to AAD; (**c**) among the 10 most increased lipids, 3 TGs, 1 DG, 1 PC, 1 PC-O, and 1 SM increased during both diet interventions, with a larger increase in the CHO diet vs. the MUFA diet for most lipid species. The blue colors represent data for MUFA vs. AAD and the orange colors represent data for CHO vs AAD (**d**) MUFA diet compared to AAD; (**e**) CHO diet compared to AAD; (**f**) among the 10 most decreased lipids, a total of 8 lipids (1 PC-O, 2 PCs, and 5 TGs) decreased during both diet interventions, with a greater decrease in the MUFA diet vs. the CHO diet for all lipids. The blue colors represent data for MUFA vs. AAD and the orange colors represent data for CHO vs AAD Abbreviations: CHO, carbohydrate; DG, diacylglycerols; Lp(a), lipoprotein(a); MUFA, monounsaturated fatty acid; PC, phosphatidylcholine; PC-O, alkylphosphatidylcholine; TG, triacylglycerol; SM, sphingomyelin.

DELTA 2: The pattern of increased vs. decreased lipid species differed between the MUFA and the CHO diets. Thus, while a majority of the 87 lipid species decreased in abundance during the MUFA diet (n = 67 lipids), the opposite result was noted for the CHO diet, with an increase in abundance for the majority of the lipid species (n = 50) (Figure 1c,d). The 10 most increased lipid species for both the MUFA and the CHO diets compared with the AAD included 6 longer-chain TG species, 1 PC, 1 PC-O, 1 SM, and 1 diacylglycerol (DG) (Table 4 and Table 5) (Figure 3a,b), which is indicative of a selective increase in longer-chain TG species in response to SFA reduction. This was in line with the overall pattern of changes seen in DELTA 1. However, out of the six TGs, only three increased during both diet periods (Figure 3c). Similarly to the DELTA 1 findings, replacement of SFA with MUFA or CHO resulted in a significant decrease in the abundance of shorter-chain TGs, as the 10 most decreased lipid species included 7 shorter-chain TGs, 2 PCs, and 1 PC-O for the MUFA diet period (Figure 3d), and 6 shorter-chain TGs, 3 PCs, and 1 PC-O for the CHO diet period (Figure 3e) (Table 5). Among the 10 most decreased lipids, a total of 8 lipids (1 PC-O, 2 PCs, and 5 TGs) decreased during both diet interventions, with a greater decrease in abundance for the MUFA diet vs. the CHO diet for all species (Figure 3f).

Intraindividual changes in Lp(a) lipid species between diet interventions are shown in heatmaps in Supplemental Figures S2 and S3. As seen in Supplemental Figure S2, a consistent pattern of increased abundance for longer-chain TGs and a decrease in abundance for shorter-chain TGs and PC species was observed for both men and women in DELTA 1. For DELTA 2, in addition to longer-chain TGs, lipids that increased included DG and longer-chain PCs (Supplemental Figure S3). In addition to shorter-chain TGs, shorter-chain PCs showed decreased abundance. Again, the pattern was consistent and similar for men and women (Supplemental Figure S3).

Furthermore, our PCA analyses of the lipidomics data showed clear differences between the diet groups in both DELTA trials (Supplemental Figure S4). In DELTA 1, the three diet groups were clearly separated, with the Step-1 diet positioned between the AAD and Low-Sat diets, consistent with the graded decrease in SFA. In DELTA 2, the three diet groups (AAD, CHO, and MUFA) also showed separation, with the most pronounced distinction observed between the AAD and MUFA diets.

We also performed exploratory analyses to assess the effects of diet, period, and diet sequence in each DELTA trial (Supplemental Tables S4 and S5). Overall, the results of these analyses were similar to those of the primary analyses. For Lp(a)-OxPL total concentration, diet sequence had an impact on DELTA 1 (Supplemental Table S4) but not on DELTA 2 (Supplemental Table S5). Among the pre-selected Lp(a) lipid species, a single TG species in each of the DELTA studies was influenced by the diet period. Overall, the results of the diet x period interaction analysis indicated that the effect of diet was consistent across study periods in both DELTA 1 and 2, providing support to the main effect of diet (Supplemental Tables S4 and S5).

### 3.4. Correlations of Lp(a) Lipidomic Changes with Changes in Plasma Lp(a) Levels

Compared to the AAD in DELTA 1, changes in the Lp(a)-OxPL total concentration, OxPC subspecies, and the sum of four major OxPC subspecies were positively correlated with Lp(a) level changes in response to the Step-1 diet and negatively correlated with Lp(a) level changes in response to the Low-Sat diet (Supplemental Table S6). For DELTA 2, while the Lp(a)-OxPL total concentration showed a similar pattern (positively correlated in the MUFA diet, negatively correlated in the CHO diet), OxPC subspecies and the sum of the four OxPC subspecies were negatively correlated with Lp(a) level changes in the MUFA diet, and negatively correlated in response to the CHO diet. Overall, the increased lipid species showed a positive correlation and decreased lipid species showed a negative correlation with Lp(a) level changes in response to diet interventions in each DELTA trial. However, a few lipid species showed a correlation in the opposite direction, indicating the complexity of intraindividual changes in lipid metabolism (Supplemental Table S6).

### 3.5. The Roles of Properties of Triacylglycerol Species in Modulating Their Responses to Dietary Saturated Fat Reduction

**Figure 4:**
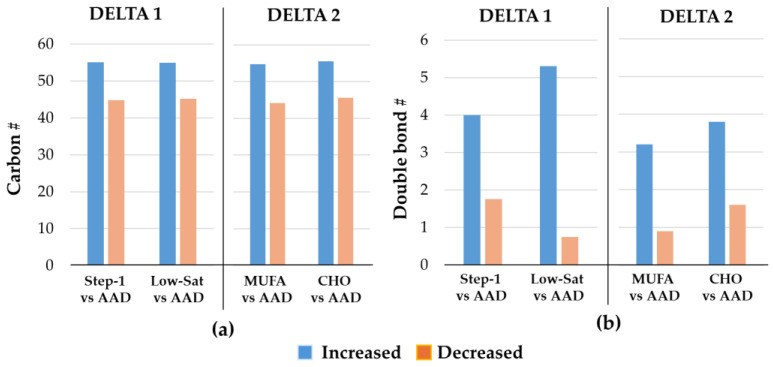
Properties of the 10 most significantly changed triacylglycerol species in DELTA studies. The bar graphs show the median numbers of (**a**) carbon atoms, and (**b**) double bonds for the 10 most increased vs. the 10 most decreased triacylglycerol species during the intervention diets compared with the average American diet (AAD), shown separately for the DELTA 1 and 2 studies. Abbreviations: CHO, carbohydrate; MUFA, monounsaturated; Sat, saturated; #, number.

As the majority of Lp(a) lipids that showed the greatest changes were either longer-chain (increased) or shorter-chain (decreased) TGs, we further tested whether the number of carbon atoms (length) or double bonds (saturation level) in the TG species would impact the degree and direction of the response. We focused on the TG species that showed the largest change in abundance, either increased or decreased, for each of the two DELTA studies. In both DELTA studies, during all intervention diets, compared to the most decreased TG species, the TG species that increased the most had a higher average number of carbon atoms (~55 vs. 45) (Figure 4a). Similarly, during all intervention diets in both DELTA studies, the TG species that increased the most in abundance had a higher average number of double bonds compared to the TG species with the largest decrease in abundance (Figure 4b). A similar pattern of modulatory role was noted for the PC class as longer-chain PCs increased, while shorter-chain PCs decreased in abundance in response to intervention diets in both DELTA trials.

## 4. Discussion

To our knowledge, this is the first study to comprehensively characterize the Lp(a) lipidome in response to diet interventions. Lowering of dietary SFA intake is recommended for LDL-C management and CVD risk prevention at the population level [27]. However, a reduction in SFA intake represents one of the few environmental factors that increase Lp(a) concentrations [28]. The changes in Lp(a) concentrations during dietary interventions offered opportunities to assess the impact on the lipid cargo carried in Lp(a), an understudied area so far. In view of this, we characterized the OxPL spectrum and performed an analysis to map the Lp(a) lipidome responses to diet interventions.

Our findings demonstrate dynamic and consistent changes in the composition of the Lp(a) lipidome, with TG species showing the most profound changes in both DELTA trials. However, a more diverse class of lipid species, including PC, SM, and DG, changed significantly in the DELTA 2 cohort. Notably, participants in the DELTA 2 study had a more pronounced metabolic burden than those in the DELTA 1 study, suggesting a potential impact of metabolic status on Lp(a) lipidomics. These findings are noteworthy considering the recent findings demonstrating an impact of elevated Lp(a)-DG and LPC species on monocyte-driven inflammation [29]. While the latter study compared participants with high vs. low Lp(a) concentrations, we found intraindividual changes (the same individual serves as his/her own control) in response to diet modifications, illustrating the dynamic nature of the Lp(a) lipid composition in response to SFA intake modifications. In the study by Dzobo et al., individuals with elevated Lp(a) presented a distinct lipidomic profile characterized by increased DGs and lysophosphatidic acid (LPA), a precursor of LPC [29]. Functional assessment of DG 40:6, DG 38:4, and LPA 18:0 found in individuals with elevated Lp(a) showed a dose-dependent increase in the secretion of proinflammatory cytokines after DG stimulation of monocytes. It is noteworthy that in our DELTA 2 study, the 10 most increased Lp(a) lipids included DG 36:2, which increased during both the CHO and MUFA diets relative to the AAD, with a greater increase with the former.

Furthermore, the most significantly changed lipids behaved consistently, depending on their saturation status and length, in both DELTA trials. Therefore, the abundance of longer-chain polyunsaturated TG species generally increased, whereas shorter-chain unsaturated TG species decreased. This is indicative of a selective Lp(a) increase in polyunsaturated fatty acids and a decrease in monounsaturated or saturated fatty acids in response to dietary SFA modulation. The potential implications of these changes for Lp(a) particle function and atherogenicity, as well as for Lp(a)’s status in the broader lipid profile relevant to CVD risk, remain to be determined. The lack of available data on the responses of the Lp(a)-lipidome profile, including TG fatty acid properties, to diet modulation limits our ability to make any direct comparison. However, our recent study in an animal model, although based on the plasma lipidome profile, showed that an opposite feeding pattern, i.e., a high-fat diet (HFD), significantly impacted the TG species [30]. Consistent with the current results, the effect of an HFD was pronounced for TG species and depended on the degree of unsaturation of fatty acid chains. Thus, an HFD increased TGs with a low number of double bonds but decreased those with a high number of double bonds. Together, these results provide further support for the evidence that the metabolic environment (e.g., diet) can impact individual TG species, including those in Lp(a), which may substantially differ in their metabolic effects. Low SFA diets are often associated with reduced hepatic de novo lipogenesis due to a lesser production of SFA and an altered fatty acid pool with a shift toward MUFA and PUFA [30,31]. The latter results in an increased incorporation of oleic acid, linoleic acid, and eicosapentaenoic/docosahexaenoic into TGs [32]. Furthermore, low SFA diets are associated with improved lipid oxidation due to more unsaturated fats promoting mitochondrial beta-oxidation and anti-inflammatory plasma lipidome changes, where more PUFA-rich TG species contribute to reducing inflammation and improving insulin signaling [32,33,34].

A previous study compared the fatty acid composition in Lp(a) to LDL, showing a higher content of unsaturated fatty acids and lower linolenic acid, as well as the absence of eicosapentaenoic acid in Lp(a)-TG compared to LDL-TG [35]. Our findings of dynamic Lp(a)-TG changes in response to the diet raise the issue of whether this may also be the case for the LDL lipidome. Therefore, our study sets the stage for further mechanistic investigation into the relevance of Lp(a)-lipidomic changes for disease development. The potential use of individual lipidome biomarker(s), as well as summary measurements (such as risk scores) in clinical settings, needs to be addressed in future studies focused on Lp(a).

Studies have shown that the majority of proinflammatory and proatherogenic OxPL in plasma is carried by Lp(a) [5,19,20], and it has been suggested that these OxPLs contribute to CVD risk [6]. Faghihnia et al. reported higher concentrations of Lp(a) and OxPL-apo(a) following a low-fat, high-CHO diet compared with a high-fat, low-CHO diet [21]. In contrast, our study did not find significant changes in Lp(a)-OxPL total concentrations during SFA reduction in the DELTA 1 study. However, in DELTA 2, the concentration of the four major Lp(a)-OxPL subspecies decreased significantly following the CHO diet when compared to AAD, and the change was primarily driven by a significant decrease observed for ALDOPC. Although differences in study design and methodology might contribute to the differing findings, it should be noted that this is an emerging field, and further studies are needed. In this context, the findings in the study by van der Valk et al. [23] in multiple settings (in vivo, ex vivo, and in vitro) shed light on the role of OxPL in mediating Lp(a) atherothrombosis. The study showed that individuals with elevated Lp(a) had increased arterial inflammation and enhanced peripheral blood mononuclear cells trafficking to the arterial wall compared to individuals with normal Lp(a) levels. In addition, monocytes isolated from individuals with high Lp(a) remain in a long-lasting primed state, as evidenced by an increased capacity to transmigrate and produce proinflammatory cytokines on stimulation. In vitro studies showed that OxPL contained in Lp(a) augments the proinflammatory response in monocytes, and this effect was attenuated by inactivating Lp(a)-OxPL or removing OxPL from apo(a) [23]. These findings suggested that Lp(a) induces monocyte trafficking to the arterial wall and mediates proinflammatory responses through its OxPL content. Therefore, studies aimed at evaluating the clinical significance of the observed reduction in Lp(a)-OxPL concentration, in particular ALDOPC, in response to dietary SFA reduction in metabolically challenged at-risk individuals are warranted. In view of this, contextualizing the observed lipidomic changes in Lp(a) within broader metabolic networks may help identify specific diet-affected synthesis and/or catabolism pathways.

Furthermore, in both DELTA trials, we previously studied hemostatic/thrombogenic factors and found that replacing dietary SFA with carbohydrate decreased factor VIIc and increased fibrinogen in both healthy and metabolically unhealthy individuals [16]. Similarly, replacing SFA with MUFA decreased factor VIIc and increased fibrinogen, but to a lesser extent than when replaced with carbohydrate. On the other hand, PAI-1 increased in healthy individuals but not in metabolically challenged individuals [16]. These directionally opposite results underscore the complexity of dietary modulation of cardiometabolic risk and the need for a comprehensive assessment that takes multiple factors into account.

On an equimolar basis, Lp(a) has been shown to be five to six times more atherogenic than LDL [36]. Lp(a) particles can enter the vessel wall and initiate inflammation, plaque formation, and vascular disease progression through their OxPL [5,37] and lipid [29] components. However, relatively scant attention has been paid to the composition of the Lp(a) lipid portion, part of the cargo subject to uptake by the vessel wall. It is notable that much of the lipid cargo was affected by diet regimens. Shorter-chain triacylglycerols and phosphatidylcholines were replaced by longer-chain variants in response to SFA reduction. To what extent such modifications might affect Lp(a)’s atherogenic potential remains to be determined, but it is likely that the type of lipid species subject to delivery to the vessel wall via lipoproteins will depend on diet composition. While mechanisms underlying dietary SFA reduction-induced increases in Lp(a) levels remain poorly understood, it likely results from an increased hepatic apo(a) synthesis rather than a reduced catabolism of Lp(a). Supporting this view, an early randomized crossover trial among cynomolgus monkeys, whose Lp(a) has similar immunologic properties to humans [38], showed that a MUFA replacement for SFA resulted in changes in Lp(a) levels as well as in hepatic apo(a) mRNA abundance [39]. These findings suggested an impact on apo(a) transcription and that dietary fatty acids regulate hepatic apo(a) synthesis, affecting Lp(a) levels. Notably, Lp(a) has a limited species distribution and has only been detected in humans, nonhuman primates, and hedgehogs [40,41,42,43].

Recently, we discussed new evidence related to current dietary recommendations to reduce SFA intake in modulating an individual’s global risk of CVD [44]. Growing evidence from well-controlled clinical studies suggests that a reduction in dietary SFA intake increases Lp(a) levels while lowering LDL-C concentrations. While the clinical relevance and/or the exact contribution of this discordant response in Lp(a) and LDL-C to CVD risk remains to be established, it is likely that any net effect on CVD risk will be impacted by the relative balance between LDL-C and Lp(a) in a given individual. The opposite effect of the SFA reduction on these two highly atherogenic lipoproteins raises concerns regarding current dietary recommendations to reduce SFA intake and reinforces the concept of precision nutrition and implementation of individualized dietary guidance. To date, insufficient evidence exists to make dietary recommendations for patients with elevated Lp(a) levels; however, our novel findings on the dynamic nature of the Lp(a) lipidome continue to reinforce the need for addressing the potential challenges of dietary SFA in reducing CVD risk in diverse patient and population groups.

Our study has some strengths and limitations. While many studies have focused on the *LPA* gene and the contribution of genetic variability to Lp(a) concentrations [1], the present findings emphasize the dynamic nature of the Lp(a) lipid composition. In particular, the results demonstrate that Lp(a) particle properties in any given individual can be affected by diet interventions within the regular food spectrum. However, due to method optimization restrictions and attempts to include as many lipids as possible, cholesterol and cholesterol esters, which are prone to in-source fragmentation and have poor ionization efficiency [45,46,47], were not considered in the lipidomics analysis. Thus, future studies targeting this area are warranted. Furthermore, while we report findings in the two DELTA studies, we recognize differences in the study cohorts and intervention diets between the two studies. Due to our focus on the Lp(a) lipidome, studies focusing on intraindividual LDL-lipidome responses to diet interventions will be required to assess differences and similarities in the responses of Lp(a) vs. LDL lipid compositions. The small number of African American participants in our two study cohorts limited our ability to consider ethnicity/race in our analyses. Given the well-known differences in Lp(a) levels across ethnicities [48,49,50,51,52], larger studies with diverse groups of individuals are needed to confirm the findings. In addition, these studies should consider assessing potential roles of genetic variants such as the apo(a) size polymorphism as modulators of dietary responses. The averages of replicate measurements for the endpoints taken at the end of the AAD or the intervention diets were used for analyses. This approach allowed a new steady state for Lp(a) as a total of 11 to 14 weeks between the compared timepoints, helping to minimize confounding by the randomization order and the carryover effect. Finally, although 7–8 weeks of controlled feeding is a substantial period of time, this is relatively brief compared to lifelong dietary habits and limits assessments of any long-term clinical outcomes. However, the intervention diets represented diets recommended for the population at large (specifically reduced saturated fat intake) [53,54,55].

## 5. Conclusions

Replacing dietary SFA with complex carbohydrates or MUFA resulted in changes in Lp(a) particle composition, with the most pronounced changes being within the triacylglycerol species. Specifically, reducing SFA intake resulted in an increase in the relative abundance of longer-chain polyunsaturated triacylglycerol species, while shorter-chain saturated or monounsaturated triacylglycerol species were reduced. Additionally, the findings suggest that SFA reduction, in particular replacement with carbohydrates, may modulate Lp(a)’s OxPL composition in metabolically at-risk individuals who have Lp(a) levels within the normal range.

Presently, clinical guidelines estimate Lp(a)-attributable risk based on plasma Lp(a) levels, and dietary guideline-recommended interventions, i.e., lowering of dietary SFA intake, significantly increase Lp(a) levels. In some cases, a SFA reduction-induced increases in Lp(a) levels result in a substantial upward shift in the distribution of Lp(a)-attributable CVD risk categories. However, how these SFA reduction-induced changes in Lp(a) lipidomic properties, as we described, may impact the Lp(a) level-based cardiovascular risk estimation for a given individual remains to be determined.

Taken together, these findings underscore the dynamic nature of the Lp(a) particle, and further studies are needed to explore the role of Lp(a) lipidome in atherogenicity and/or cardiovascular risk.

## Figures and Tables

**Table 1:** Plasma Lp(a) and Lp(a)-OxPL total concentrations and subspecies abundance at the end of each dietary intervention in DELTA 1 and 2.

Variables	DELTA 1 (n = 96)			DELTA 2 (n = 79)		
	AAD	Step-1	Low-Sat	AAD	MUFA	CHO
Lp(a) concentration (mg/dL)	11.0 (5.0; 30.2)	13.0 (5.0; 31.1)	14.0 (6.0; 34.8)	7.0 (2.0; 17.0)	9.0 (3.0; 22.0)	7.5 (3.0; 22.0)
Lp(a)-OxPL total concentration (U/L)	8.3 (5.7; 10.5)	7.5 (5.3; 10.3)	8.0 (5.4; 9.7)	10.1 (7.3; 12.6)	7.7 (6.1; 12.2)	10.0 (7.0; 12.1)
Lp(a)-OxPC subspecies (g/U):						
ALDOPC	1.05 (0.34; 3.82)	1.05 (0.27; 3.17)	0.95 (0.23; 2.69)	1.29 (0.38; 4.10)	1.12 (0.33; 3.43)	0.88 (0.37; 3.43) ^a^
POVPC	0.24 (0.07; 0.70)	0.17 (0.06; 0.60)	0.18 (0.05; 0.67)	0.21 (0.08; 0.88)	0.19 (0.07; 0.72)	0.23 (0.10; 0.75)
PAzPC	0.10 (0.02; 0.40)	0.08 (0.03; 0.31)	0.08 (0.03; 0.27)	0.13 (0.02; 0.33)	0.09 (0.03; 0.29)	0.11 (0.03; 0.32)
PGPC	0.02 (0.01; 0.05)	0.01 (0.01; 0.06)	0.02 (0.00; 0.06)	0.02 (0.01; 0.05)	0.02 (0.01; 0.05)	0.01 (0.00; 0.06)
Sum of four OxPCs	1.52 (0.43; 4.97)	1.36 (0.36; 4.09)	1.22 (0.32; 3.70)	1.58 (0.51; 5.53)	1.59 (0.42; 4.48)	1.20 (0.49; 4.63) ^b^

Data are median (interquartile range). ^a^ Significantly decreased in CHO diet when compared to AAD in DELTA 2 (*p* = 0.014). ^b^ Significantly decreased in CHO diet when compared to AAD in DELTA 2 (*p* = 0.028). Abbreviations: AAD, average American diet; ALDOPC, 1-palmitoyl-2-(9-oxo-nonanoyl)-sn-glycero-3-phosphocholine; CHO, carbohydrate diet; OxPL, oxidized phospholipids; PAzPC, 1-palmitoyl-2-azelaoyl-sn-glycero-3-phosphocholine, PGPC, 1-palmitoyl-2-glutaryl-sn-glycero-3-phosphocholine, and POVPC, 1-palmitoyl-2-(5′-oxo-valeroyl)-sn-glycero-3-phosphocholine.

**Table 2:** Ten most significantly increased or decreased Lp(a)-specific lipids during the Step-1 diet compared to the average American diet in DELTA 1.

	FC ^b^	Log_2_FC ^b^	*p*-Value	*p*-Value Adjusted ^c^
Most increased lipids ^a^				
PC 40:7 B	1.35	0.43	6.11× 10^−6^	0.014
TG 54:2 B|18:0_18:1_18:1	1.32	0.40	3.43 × 10^−8^	0.008
TG 56:5|16:0_18:1_22:4	1.34	0.42	5.78 × 10^−4^	0.038
TG 55:6	1.34	0.42	1.33 × 10^−3^	0.048
TG 54:3|18:1_18:1_18:1	1.36	0.45	4.65 × 10^−9^	0.005
TG 54:4 A|18:1_18:1_18:2	1.37	0.46	2.86 × 10^−8^	0.007
TG 56:3 B|18:1_18:1_20:1	1.38	0.46	1.26 × 10^−5^	0.018
TG 56:4 B|18:0_18:0_20:4	1.38	0.47	4.09 × 10^−6^	0.013
TG 56:4 A|18:1_18:2_20:1	1.39	0.48	4.90 × 10^−6^	0.014
TG 54:5 A|18:1_18:2_18:2	1.45	0.54	2.61 × 10^−6^	0.011
Most decreased lipids ^a^				
PC 28:0	0.32	−1.64	4.62 × 10^−12^	0.003
PC 30:2	0.28	−1.82	2.11 × 10^−9^	0.005
TG 48:4 A|14:0_16:0_18:4	0.50	−0.99	8.63 × 10^−5^	0.025
TG 46:4|10:0_18:2_18:2	0.48	−1.07	2.02 × 10^−4^	0.028
TG 46:1 B|14:0_14:0_18:1	0.38	−1.41	2.12 × 10^−6^	0.011
TG 46:1 A|12:0_16:1_18:0	0.32	−1.64	2.88 × 10^−5^	0.019
TG 44:0|12:0_16:0_16:0	0.29	−1.77	1.49 × 10^−6^	0.010
TG 44:1|12:0_14:0_18:1	0.26	−1.94	2.92 × 10^−5^	0.020
TG 42:1|12:0_12:0_18:1	0.15	−2.76	1.27 × 10^−3^	0.047
TG 42:2|08:0_16:1_18:1	0.10	−3.38	5.10 × 10^−5^	0.023

^a^ Annotations A and B represent isomers. ^b^ Fold changes were estimated as described in the methods section and log_2_ transformed to demonstrate the direction of effect. ^c^ Adjusted for false discovery rate. Abbreviations: FC, fold change; PC, phosphatidylcholine; TG, triacylglycerol.

**Table 3:** Ten most significantly increased or decreased Lp(a)-specific lipids during the Low-Sat diet compared to the average American diet in DELTA 1.

	FC ^b^	Log_2_FC ^b^	*p*-Value	*p*-Value Adjusted ^c^
Most increased lipids ^a^				
TG 54:3|18:1_18:1_18:1	1.51	0.59	1.32 × 10^−15^	0.007
TG 56:8 A|18:2_18:2_20:4	1.52	0.61	1.59 × 10^−8^	0.034
TG 56:9|18:2_18:2_20:5	1.54	0.62	5.53 × 10^−7^	0.042
TG 54:5 B|18:1_18:2_18:2	1.55	0.63	1.68 × 10^−12^	0.014
TG 54:4 A|18:1_18:1_18:2	1.58	0.66	1.95 × 10^−16^	0.005
TG 55:6	1.61	0.68	2.06 × 10^−9^	0.025
TG 56:4 A|18:1_18:2_20:1	1.67	0.74	1.09 × 10^−13^	0.010
TG 54:5 A|18:1_18:2_18:2	1.69	0.75	1.88 × 10^−13^	0.011
TG 56:3 B|18:1_18:1_20:1	1.69	0.76	2.11 × 10^−9^	0.026
TG 54:6 A|18:1_18:2_18:3	1.83	0.88	1.08 × 10^−7^	0.038
Most decreased lipids ^a^				
PC 33:1 B	0.35	−1.51	5.94 × 10^−23^	0.003
PC 28:0	0.16	−2.65	5.65 × 10^−16^	0.007
PC 30:2	0.15	−2.78	6.86 × 10^−13^	0.013
PC-O 30:0	0.42	−1.25	3.16 × 10^−24^	0.002
PC-O 30:1	0.28	−1.81	2.18 × 10^−25^	0.001
TG 46:3	0.42	−1.25	3.36 × 10^−9^	0.027
TG 46:1 B|14:0_14:0_18:1	0.25	−2.02	3.2 × 10^−10^	0.020
TG 46:1 A|12:0_16:1_18:0	0.21	−2.23	1.66 × 10^−7^	0.038
TG 44:0|12:0_16:0_16:0	0.17	−2.55	4.97 × 10^−9^	0.030
TG 44:1|12:0_14:0_18:1	0.16	−2.68	3.9 × 10^−7^	0.041

^a^ Annotations A and B represent isomers. ^b^ Fold changes were estimated as described in the methods section and log2 transformed to demonstrate the direction of effect. ^c^ Adjusted for false discovery rate. Abbreviations: FC, fold change; PC, phosphatidylcholine; PC-O, alkylphosphatidylcholine; TG, triacylglycerol.

**Table 4:** Ten most significantly increased or decreased Lp(a)-specific lipids during the MUFA diet compared to the average American diet in DELTA 2.

	FC ^b^	Log_2_FC ^b^	*p*-Value	*p*-Value Adjusted ^c^
Most increased lipids ^a^				
DG 36:2	1.48	0.56	9.64 × 10^−8^	0.036
PC 42:7	1.50	0.58	6.67 × 10^−9^	0.028
PC-O 40:8	1.37	0.46	4.73 × 10^−10^	0.024
SM d38:4	1.33	0.41	1.61 × 10^−6^	0.047
TG 55:3|18:0_18:2_19:1	1.37	0.45	4.48 × 10^−17^	0.008
TG 56:4 A|18:1_18:2_20:1	1.41	0.49	8.93 × 10^−9^	0.030
TG 54:4 A|18:1_18:1_18:2	1.45	0.54	5.35 × 10^−17^	0.009
TG 56:3 B|18:1_18:1_20:1	1.50	0.59	1.59 × 10^−7^	0.038
TG 54:2 B|18:0_18:1_18:1	1.53	0.61	5.64 × 10^−16^	0.011
TG 54:3|18:1_18:1_18:1	1.64	0.72	9.16 × 10^−17^	0.009
Most decreased lipids ^a^				
PC-O 30:1	0.28	−1.81	5.00 × 10^−24^	0.002
PC 30:2	0.14	−2.87	2.11 × 10^−19^	0.006
PC 28:0	0.14	−2.87	1.03 × 10^−23^	0.003
TG 46:3	0.29	−1.79	2.27 × 10^−9^	0.026
TG 46:1 B|14:0_14:0_18:1	0.20	−2.32	2.33 × 10^−11^	0.016
TG 46:1 A|12:0_16:1_18:0	0.18	−2.51	1.67 × 10^−8^	0.034
TG 44:1|12:0_14:0_18:1	0.16	−2.69	9.81 × 10^−9^	0.031
TG 44:0|12:0_16:0_16:0	0.13	−2.91	1.04 × 10^−11^	0.015
TG 42:1|12:0_12:0_18:1	0.08	−3.71	1.01 × 10^−7^	0.036
TG 42:2|08:0_16:1_18:1	0.06	−4.18	2.25 × 10^−7^	0.038

^a^ Annotations A and B represent isomers. ^b^ Fold changes were estimated as described in the methods section and log2 transformed to demonstrate the direction of effect. ^c^ Adjusted for false discovery rate. Abbreviations: DG, diacylglycerol; FC, fold change; MUFA, monounsaturated fatty acid; PC, phosphatidylcholine; PC-O, alkylphosphatidylcholine; SM, sphingomyelin; TG, triacylglycerol.

**Table 5:** Ten most significantly increased or decreased Lp(a)-specific lipids during the CHO diet compared to the average American diet in DELTA 2.

	FC ^b^	Log_2_FC ^b^	*p*-Value	*p*-Value Adjusted ^c^
Most increased lipids ^a^				
DG 36:2	1.53	0.62	4.44 × 10^−7^	0.025
PC 42:7	1.73	0.79	3.97 × 10^−11^	0.009
PC-O 44:8	1.97	0.98	1.23 × 10^−5^	0.036
SM d38:4	1.46	0.55	3.08 × 10^−5^	0.040
TG 56:4 A|18:1_18:2_20:1	1.45	0.53	6.83 × 10^−11^	0.010
TG 58:8 B	1.46	0.54	5.83 × 10^−10^	0.013
TG 54:2 B|18:0_18:1_18:1	1.48	0.57	5.92 × 10^−14^	0.006
TG 56:4 B|18:0_18:0_20:4	1.54	0.63	3.82 × 10^−6^	0.031
TG 56:2|18:0_18:1_20:1	1.58	0.66	4.1 × 10^−5^	0.042
TG 54:3|18:1_18:1_18:1	1.61	0.69	1.33 × 10^−14^	0.005
Most decreased lipids ^a^				
PC 32:3	0.41	−1.27	4.03 × 10^−11^	0.009
PC 28:0	0.19	−2.41	8.59 × 10^−21^	0.002
PC 30:2	0.15	−2.69	6.58 × 10^−17^	0.003
PC-O 30:1	0.40	−1.34	1.16 × 10^−20^	0.002
TG 46:3	0.39	−1.34	7.04 × 10^−5^	0.047
TG 48:4 A|14:0_16:0_18:4	0.39	−1.35	2.31 × 10^−8^	0.017
TG 46:1 B|14:0_14:0_18:1	0.30	−1.74	2.15 × 10^−7^	0.023
TG 46:1 A|12:0_16:1_18:0	0.26	−1.96	1.3 × 10^−5^	0.036
TG 44:1|12:0_14:0_18:1	0.22	−2.16	5.14 × 10^−5^	0.045
TG 44:0|12:0_16:0_16:0	0.19	−2.42	2.51 × 10^−8^	0.018

^a^ Annotations A and B represent isomers. ^b^ Fold changes were estimated as described in the methods section and log_2_ transformed to demonstrate the direction of effect. ^c^ Adjusted for false discovery rate. Abbreviations: CHO, carbohydrate; DG, diacylglycerol; FC, fold change; PC, phosphatidylcholine; PC-O, alkylphosphatidylcholine; SM, sphingomyelin; TG, triacylglycerol.

## Data Availability

The original contributions presented in this study are included in the article/Supplementary Materials. Further inquiries can be directed to the corresponding author.

## Supplementary Materials

The following supporting information can be downloaded at: https://www.mdpi.com/article/10.3390/nu17193113/s1, Table S1: Nutrient values for experimental diets; Table S2: Participant characteristics during the reference average American diet in the DELTA 1 and 2 trials; Table S3: Proportion of significantly altered lipids by lipid class during intervention diets in DELTA 1 and 2; Table S4: Significance levels from a linear mixed-effects model for the effects of diet, period, sequence and diet x period interaction in DELTA 1; Table S5: Significance levels from a linear mixed-effects model for the effects of diet, period, sequence, and diet x period interaction in DELTA 2; Table S6: Correlations for changes in Lp(a) levels with Lp(a)-OxPL measures and most significantly changed lipid species during intervention diets in DELTA 1 and 2; Figure S1: CONSORT chart; Figure S2: Heatmap of intraindividual changes in response to diet interventions for the most significantly changed Lp(a) lipids in male and female in DELTA 1; Figure S3: Heatmap of intraindividual changes in response to diet interventions for the most significantly changed Lp(a) lipids in male and female in DELTA 2; Figure S4: 3D scores plots from principal component analysis in DELTA 1 and 2.

## Abbreviations

The following abbreviations are used in this manuscript:

AAD	average American diet
apo(a)	apolipoprotein(a)
ALDOPC	1-palmitoyl-2-(9′-oxo-nanonoyl)-sn-glycero-3-phosphocholine
CHO	Carbohydrate
DELTA	Dietary Effects on Lipoproteins and Thrombogenic Activity
DG	diacylglycerols
FC	fold change
Lp(a)	lipoprotein(a)
Log_2_FC	log2 transformed fold change
Low-Sat	low-saturated-fat diet
LPC	lysophosphatidylcholine
OxPC	oxidized phosphocholines
OxPL	oxidized phospholipids
PAzPC	1-palmitoyl-2-azelaoyl-sn-glycero-3-phosphocholine
PC-O	alkylphosphatidylcholine
PGPC	1-palmitoyl-2-glutaryl-sn-glycero-3-phosphocholine
POVPC	1-palmitoyl-2-(5′-oxo-valeroyl)-sn-glycero-3-phosphocholine
TG	triacylglycerols
SFA	saturated fatty acids
