# Supplementary material for: Lipoprotein(a) Lipidome: Responses to Reduced Dietary Saturated Fat Intake in Two Randomized Controlled Feeding Trials
Source: Nutrients. 2025 Sep 30;17(19):3113. doi: 10.3390/nu17193113 (PMC12525883; doi:10.3390/nu17193113)
Supplement: Supplementary file 1 [file nutrients-17-03113-s001.zip › nutrients-3879548-supplementary.pdf]

SUPPLEMENTARY MATERIALS

Article

Lipoprotein(a) Lipidome: Responses to Reduced Dietary Saturated Fat Intake in Two Randomized Controlled Feeding Trials

Munkhtuya Myagmarsuren [1], Hayley G. Law [1], Wei Zhang [1], Tselmen Anuurad [1], Heejung Bang [2], Lauren M. Bishop [3], Tong Shen [3], Oliver Fiehn [3], Kristina S. Petersen [4], Lars Berglund [1] and Byambaa Enkhmaa [1,*]

[1] Department of Internal Medicine, University of California-Davis, Davis, CA, USA; mmyagmarsuren@health.ucdavis.edu; haglaw@ucdavis.edu; wzhang@health.ucdavis.edu; tanuurad@gmail.com; lberglund@ucdavis.edu;
[2] Department of Public Health Sciences, University of California-Davis, Davis, CA, USA; hbang@ucdavis.edu;
[3] West Coast Metabolomics Center, University of California-Davis, Davis, CA, USA; lbishop@ucdavis.edu; tsshen@ucdavis.edu; ofiehn@ucdavis.edu;
[4] Department of Nutritional Sciences, The Pennsylvania State University, University Park, PA 16802, USA; kup63@psu.edu;
* Correspondence: ebyambaa@health.ucdavis.edu; Tel.: +1 (530) 754-7253

Supplemental Table S1. Nutrient values for experimental diets

Nutrient	DELTA 1 [a]			DELTA 2 [b]		
	**AAD**	**Step-1 diet**	**Low-Sat diet**	**AAD**	**MUFA diet**	**CHO diet**
Protein (%E)	15	15	15	16	16	16
Carbohydrate (%E)	48	55	59	47	47	54
Total fat (%E):	37	30	26	37	37	30
SFA (%E)	16	9	5	16	8	8
MUFA (%E)	14	14	14	14	22	15
PUFA (%E)	7	7	7	7	7	7
Cholesterol (mg/d)	300	300	300	300	300	300

[a] Step-1 diet, 7% of energy from saturated fats replaced with carbohydrates; Low-Sat diet, 11% of % energy from saturated fats replaced with carbohydrates. [b] MUFA diet, 7% of energy from saturated fats replaced with monounsaturated fatty acids; CHO diet, 7% of energy from saturated fats replaced with carbohydrates. Abbreviations: AAD, average American diet; %E, percent energy; SFA, saturated fatty acids; MUFA, monounsaturated fatty acids; PUFA, polyunsaturated fatty acids.

Supplemental Table S2. Participant characteristics during the reference average American diet in the DELTA 1 and 2 trials

Variables	DELTA 1 [a]	DELTA 2 [a]
Number of participants (n)	96	79
Age (years)	38 ± 14	35 ± 9
Female, n (%)	52 (54%)	27 (34%)
Black participants, n (%)	25 (26%)	8 (10%)
Body mass index (kg/m^2)	24.5 ± 3.2	27.7 ± 4.5
LDL cholesterol (mg/dL)	131 ± 27	126 ± 22
HDL cholesterol (mg/dL)	52 ± 11	41 ± 9
Lipoprotein(a) (mg/dL)	11.0 (5.0; 30.2)	7.0 (2.0; 17.0)
Triglycerides (mg/dL)	86 (62; 112)	124 (95; 164)

[a] The lipid values (mg/dL) are shown as mean ± SD for LDL cholesterol and HDL cholesterol and median (interquartile range) for lipoprotein(a) and triglycerides.

Supplemental Table S3. Proportion of significantly altered lipids by lipid class during intervention diets in DELTA 1 and 2

Lipid classes (annotations)	# of subspecies [a]	DELTA 1						DELTA 2					
		Step-1 vs AAD			Low-Sat vs AAD			MUFA vs AAD			CHO vs AAD		
		Total Sig.	↑	↓	Total Sig.	↑	↓	Total Sig.	↑	↓	Total Sig.	↑	↓
Triacylglycerol (TG)	90	44.4	28.9	15.6	48.9	33.3	15.6	31.1	8.9	22.2	42.2	26.7	15.6
Sphingomyelin (SM)	83	10.8	4.8	6.0	12.0	1.2	10.8	13.3	2.4	10.8	13.3	7.2	6.0
Phosphatidylcholine (PC)	74	28.4	17.6	10.8	24.3	8.1	16.2	31.1	5.4	25.7	24.3	8.1	16.2
Alkylphosphatidylcholine (PC O)	73	6.8	4.1	2.7	5.5	0.0	5.5	9.6	2.7	6.8	13.7	11.0	2.7
Lysophosphatidylcholine (LPC)	25	16.0	12.0	4.0	8.0	0.0	8.0	24.0	4.0	20.0	12.0	4.0	8.0
Ceramide (Cer)	16	0.0	0.0	0.0	6.3	0.0	6.3	18.8	0.0	18.8	0.0	0.0	0.0
Diacylglycerol (DG)	14	21.4	14.3	7.1	28.6	21.4	7.1	28.6	7.1	21.4	14.3	7.1	7.1
Acylcarnitine (CAR)	11	0.0	0.0	0.0	9.1	0.0	9.1	0.0	0.0	0.0	9.1	0.0	9.1

Data are shown as percentage (%) of significantly changed (total, increased, or decreased) lipids in each of the eight largest lipid class during intervention diets. The upward arrow indicates the % increase and the downward arrow indicates the % decrease. False discovery rate adjusted significance level was assessed at $p<0.05$. [a] Denotes number of subspecies in each lipid class.

Supplemental Table S4. Significance levels from a linear mixed-effects model for the effects of diet, period, sequence, and diet x period interaction in DELTA 1 [a]

Variable	Diet		Period [b]		Diet sequence [c]					Diet x period [d]			
	Step-1 vs AAD	Low-Sat vs AAD	2	3	ACB	BAC	BCA	CAB	CBA	B:2	B:3	C:2	C:3
Lp(a) (mg/dL) [e]	0.001	0.038	0.096	0.297	0.185	0.493	0.529	0.582	0.521	0.021	0.792	0.654	0.029
Total Lp(a)-OxPL (U/L) [e]	0.999	0.682	0.751	0.255	0.178	0.020	0.036	0.071	0.097	0.998	0.110	0.490	0.214
Sum of 4 OxPC subspecies (g/U) [e]	0.605	0.662	0.967	0.995	0.648	0.500	0.523	0.684	0.936	0.866	0.671	0.347	0.292
PC 28:0	0.000	<.0001	0.937	0.028	0.307	0.689	0.810	0.341	0.801	0.189	0.125	0.684	0.154
PC 30:2	0.001	0.001	0.888	0.038	0.388	0.905	0.860	0.470	0.460	0.293	0.130	0.602	0.149
TG 44:0	0.009	0.010	0.954	0.024	0.545	0.883	0.562	0.601	0.522	0.561	0.322	0.979	0.087
TG 44:1	0.015	0.057	0.930	0.054	0.746	0.708	0.779	0.461	0.389	0.470	0.463	0.767	0.088
TG 46:1 A	0.008	0.052	0.866	0.244	0.582	0.663	0.741	0.280	0.216	0.574	1.000	0.586	0.168
TG 46:1 B	0.009	0.015	0.851	0.012	0.782	0.675	0.814	0.703	0.377	0.500	0.312	0.706	0.063
TG 54:3	0.005	0.001	0.286	0.707	0.640	0.740	0.674	0.514	0.359	0.869	0.866	0.014	0.622
TG 56:4 A	0.027	0.001	0.171	0.864	0.659	0.802	0.404	0.553	0.696	0.805	0.702	0.015	0.492

P-values are from a linear mixed effects model with a random intercept for each participant. Diet, period, and diet sequence were modeled as fixed effects. Eight lipid species were selected as they were consistently changed across all intervention diets in both trials. [a] Unadjusted *p*-values are shown due to the exploratory nature of the analyses. [b] Compared to randomly assigned period 1. [c] Compared to the diet sequence ABC (reference group), where A is AAD, B is the Step-1 diet, and C is the low-saturated fat diet. [d] Diet and period interaction compared to the reference diet and period (A:1,2,3; B:1; C:1), [e] *P*-values are based on square-root transformed data. Abbreviations: AAD, Average American Diet; Step-1, Step-1 diet; Lp(a), lipoprotein(a); Low-Sat, low-saturated fat diet; OxPL, oxidized phospholipid; OxPC, oxidized phosphatidylcholine; PC, phosphatidylcholine, TG, triacylglycerol.

Supplemental Table S5. Significance levels from a linear mixed-effects model for the effects of diet, period, sequence, and diet x period interaction in DELTA 2 [a]

**Variable**	**Diet**		**Period** [b]		**Diet sequence** [c]					**Diet x period** [d]			
	MUFA vs AAD	CHO vs AAD	2	3	DFE	EDF	EFD	FDE	FED	E:2	E:3	F:2	F:3
Lp(a) (mg/dL) [e]	0.966	0.101	0.058	0.986	0.398	0.186	0.344	0.207	0.997	0.030	0.279	0.722	0.997
Total Lp(a)-OxPL (U/L) [e]	0.954	0.971	0.639	0.974	0.737	0.680	0.583	0.816	0.902	0.285	0.979	0.890	0.999
Sum of 4 OxPC subspecies (g/U) [e]	0.031	0.241	0.479	0.399	0.369	0.243	0.679	0.261	0.311	0.040	0.279	0.855	0.693
PC 28:0	<.0001	<.0001	0.413	0.138	0.438	0.227	0.374	0.471	0.863	0.919	0.724	0.326	0.186
PC 30:2	<.0001	<.0001	0.434	0.126	0.326	0.387	0.629	0.919	0.777	0.768	0.402	0.277	0.202
TG 44:0	<.0001	<.0001	0.241	0.541	0.457	0.506	0.817	0.843	0.394	0.608	0.717	0.062	0.340
TG 44:1	0.001	0.0001	0.196	0.526	0.495	0.626	0.938	0.709	0.271	0.601	0.698	0.035	0.316
TG 46:1 A	0.001	0.0001	0.109	0.706	0.745	0.645	0.678	0.661	0.221	0.549	0.973	0.039	0.459
TG 46:1 B	0.0001	<.0001	0.235	0.882	0.415	0.428	0.568	0.938	0.409	0.716	0.953	0.062	0.574
TG 54:3	0.001	<.0001	0.075	0.628	0.679	0.281	0.663	0.605	0.392	0.144	0.220	0.519	0.388
TG 56:4 A	0.151	<.0001	0.103	0.786	0.363	0.501	0.822	0.891	0.443	0.251	0.246	0.882	0.049

P-values are from a linear mixed effects model with a random intercept for each participant. Diet, period, and diet sequence were modeled as fixed effects. Eight lipid species were selected as they were consistently changed across all intervention diets in both trials. [a] Unadjusted *p*-values are shown due to the exploratory nature of the analyses. [b] Compared to randomly assigned period 1. [c] Compared to the diet sequence DEF (reference group), where D is AAD, E is the MUFA diet, and F is the CHO diet. [d] Diet and period interaction compared to the reference diet and period (D:1,2,3; E:1; F:1), [e] *P*-values are based on square-root transformed data. Abbreviations: AAD, Average American Diet; MUFA, monounsaturated fat diet; CHO, carbohydrate diet; Lp(a), lipoprotein(a); OxPL, oxidized phospholipid; OxPC, oxidized phosphatidylcholine; PC, phosphatidylcholine, TG, triacylglycerol.

Supplemental Table S6. Correlations for changes in Lp(a) levels with Lp(a)-OxPL measures and most significantly changed lipid species during intervention diets in DELTA 1 and 2

(a) DELTA 1

**Step-1 vs AAD**	**Correlation coefficients**	***p*-values**	**Low-Sat vs AAD**	**Correlation coefficients**	***p*-values**
		Lp(a)-OxPL concentration			
Lp(a)-OxPL total concentration (U/L)	0.09	0.405	Lp(a)-OxPL total concentration (U/L)	-0.06	0.533
ALDOPC (g/U)	0.00	0.988	ALDOPC (g/U)	-0.03	0.736
POVPC (g/U)	0.09	0.397	POVPC (g/U)	-0.07	0.504
PAzPC (g/U)	0.07	0.474	PAzPC (g/U)	-0.06	0.549
PGPC (g/U)	0.10	0.344	PGPC (g/U)	-0.08	0.440
Sum 4 OxPCs (g/U)	0.02	0.824	Sum 4 OxPCs (g/U)	-0.05	0.652
		Most increased lipids [a]			
PC 40:7 B	-0.06	0.566	TG 54:3|18:1_18:1_18:1	0.09	0.388
TG 54:2 B|18:0_18:1_18:1	0.02	0.845	TG 56:8 A|18:2_18:2_20:4	0.08	0.448
TG 56:5|16:0_18:1_22:4	0.00	0.978	TG 56:9|18:2_18:2_20:5	0.07	0.490
TG 55:6	0.03	0.796	TG 54:5 B|18:1_18:2_18:2	0.09	0.373
TG 54:3|18:1_18:1_18:1	0.02	0.869	TG 54:4 A|18:1_18:1_18:2	0.10	0.338
TG 54:4 A|18:1_18:1_18:2	0.01	0.922	TG 55:6	0.07	0.487
TG 56:3 B|18:1_18:1_20:1	-0.02	0.813	TG 56:4 A|18:1_18:2_20:1	0.07	0.521
TG 56:4 B|18:0_18:0_20:4	-0.04	0.669	TG 54:5 A|18:1_18:2_18:2	0.10	0.319
TG 56:4 A|18:1_18:2_20:1	-0.01	0.918	TG 56:3 B|18:1_18:1_20:1	0.06	0.552
TG 54:5 A|18:1_18:2_18:2	0.01	0.889	TG 54:6 A|18:1_18:2_18:3	0.09	0.363
		Most decreased lipids [a]			
PC 28:0	-0.04	0.700	PC 33:1 B	-0.08	0.425
PC 30:2	-0.09	0.405	PC 28:0	-0.27	0.008
TG 48:4 A|14:0_16:0_18:4	-0.02	0.860	PC 30:2	-0.26	0.011
TG 46:4|10:0_18:2_18:2	-0.04	0.697	PC O-30:0	-0.13	0.212
TG 46:1 B|14:0_14:0_18:1	-0.02	0.869	PC O-30:1	-0.12	0.242
TG 46:1 A|12:0_16:1_18:0	0.02	0.813	TG 46:3	-0.21	0.037
TG 44:0|12:0_16:0_16:0	-0.02	0.829	TG 46:1 B|14:0_14:0_18:1	-0.24	0.018
TG 44:1|12:0_14:0_18:1	-0.02	0.867	TG 46:1 A|12:0_16:1_18:0	-0.23	0.028

TG 42:1 ǀ 12:0_12:0_18:1	-0.01	0.949	TG 44:0 ǀ 12:0_16:0_16:0	-0.24	0.016
TG 42:2 ǀ 08:0_16:1_18:1	-0.10	0.341	TG 44:1 ǀ 12:0_14:0_18:1	-0.30	0.003

(b) DELTA 2

MUFA vs AAD	Correlation coefficients	*p*-values	CHO vs AAD	Correlation coefficients	*p*-values
			Lp(a)-OxPL concentration		
Lp(a)-OxPL total concentration (U/L)	0.25	0.028	Lp(a)-OxPL total concentration (U/L)	-0.17	0.135
ALDOPC (g/U)	-0.04	0.740	ALDOPC (g/U)	0.06	0.610
POVPC (g/U)	-0.04	0.737	POVPC (g/U)	0.01	0.939
PAzPC (g/U)	-0.05	0.699	PAzPC (g/U)	0.01	0.926
PGPC (g/U)	0.05	0.686	PGPC (g/U)	0.03	0.765
Sum 4 OxPCs (g/U)	-0.04	0.722	Sum 4 OxPCs (g/U)	0.05	0.665
			Most increased lipids [a]		
DG 36:2	-0.15	0.191	DG 36:2	-0.04	0.749
PC 42:7	0.06	0.635	PC 42:7	0.02	0.857
PC O-40:8	0.05	0.643	PC O-44:8	0.12	0.308
SM d38:4	0.15	0.203	SM d38:4	0.04	0.711
TG 55:3 ǀ 18:0_18:2_19:1	-0.02	0.863	TG 56:4 A ǀ 18:1_18:2_20:1	-0.01	0.961
TG 56:4 A ǀ 18:1_18:2_20:1	-0.01	0.930	TG 58:8 B	0.03	0.797
TG 54:4 A ǀ 18:1_18:1_18:2	0.03	0.819	TG 54:2 B ǀ 18:0_18:1_18:1	0.04	0.717
TG 56:3 B ǀ 18:1_18:1_20:1	0.06	0.620	TG 56:4 B ǀ 18:0_18:0_20:4	0.04	0.759
TG 54:2 B ǀ 18:0_18:1_18:1	0.00	0.970	TG 56:2 ǀ 18:0_18:1_20:1	0.03	0.784
TG 54:3 ǀ 18:1_18:1_18:1	0.01	0.906	TG 54:3 ǀ 18:1_18:1_18:1	0.05	0.676
			Most decreased lipids [a]		
PC O-30:1	0.02	0.857	PC 32:3	0.18	0.123
PC 30:2	-0.04	0.733	PC 28:0	0.14	0.213
PC 28:0	-0.05	0.682	PC 30:2	0.04	0.717
TG 46:3	-0.06	0.630	PC O-30:1	0.16	0.169
TG 46:1 B ǀ 14:0_14:0_18:1	-0.06	0.587	TG 46:3	0.01	0.913
TG 46:1 A ǀ 12:0_16:1_18:0	-0.02	0.856	TG 48:4 A ǀ 14:0_16:0_18:4	0.04	0.749
TG 44:1 ǀ 12:0_14:0_18:1	-0.04	0.723	TG 46:1 B ǀ 14:0_14:0_18:1	-0.02	0.840

TG 44:0|12:0_16:0_16:0	-0.08	0.492	TG 46:1 A|12:0_16:1_18:0	0.03	0.808
TG 42:1|12:0_12:0_18:1	-0.01	0.913	TG 44:1|12:0_14:0_18:1	0.00	0.996
TG 42:2|08:0_16:1_18:1	0.00	0.997	TG 44:0|12:0_16:0_16:0	-0.07	0.527

Values represent Pearson's correlation coefficient (r) with corresponding *p*-values between unit changes in plasma Lp(a) level, Lp(a)-OxPL concentration and subspecies, and most significantly (FDR adjusted) changed (increased or decreased) lipids. Positive r values indicate a positive association and negative r values indicate an inverse association. [a] Absolute changes in Lp(a) levels were correlated with log_2-transformed fold changes of the lipid subspecies as described in the methods section.

Supplemental Figure S1. CONSORT chart

(a) DELTA 1

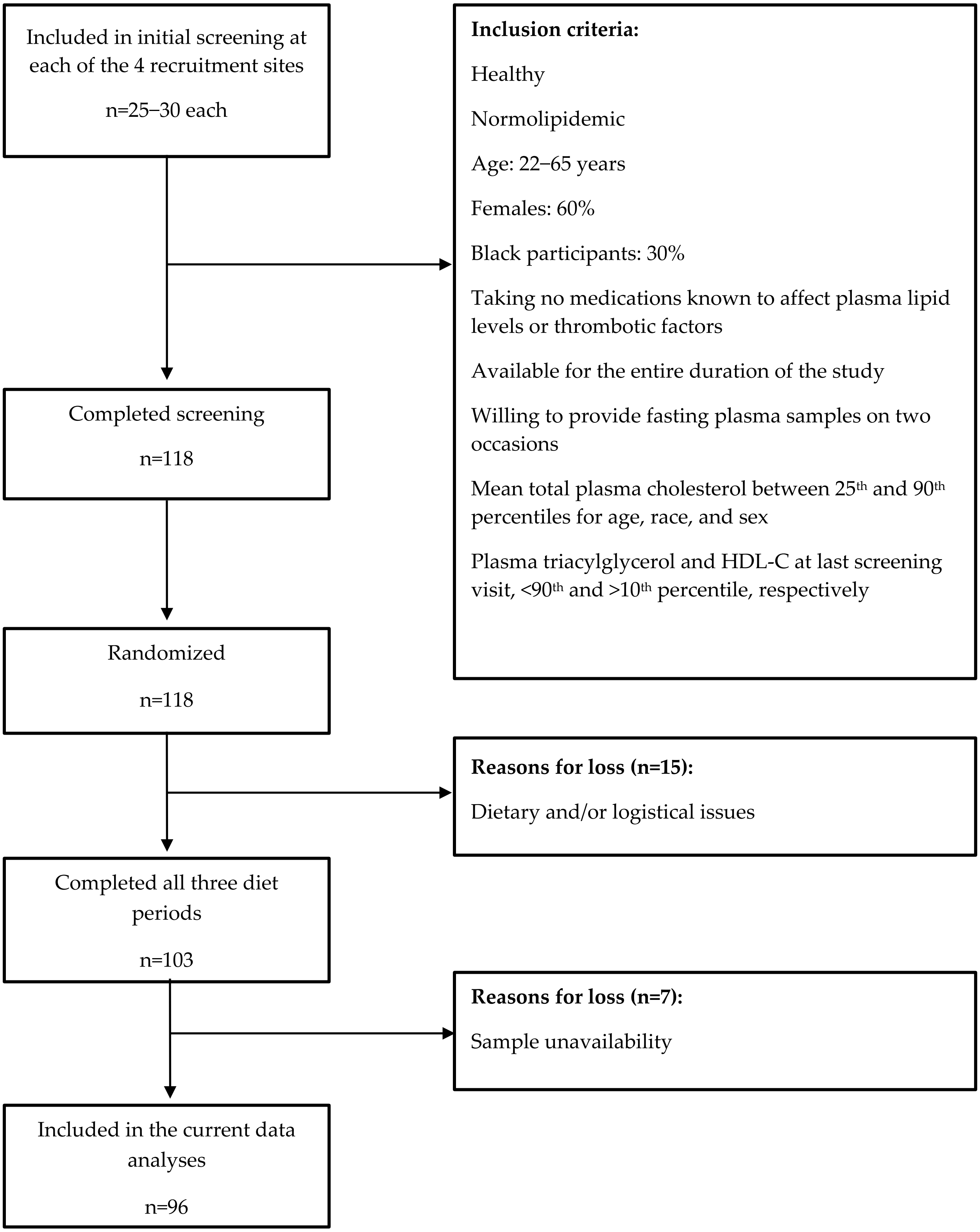

(b) DELTA 2

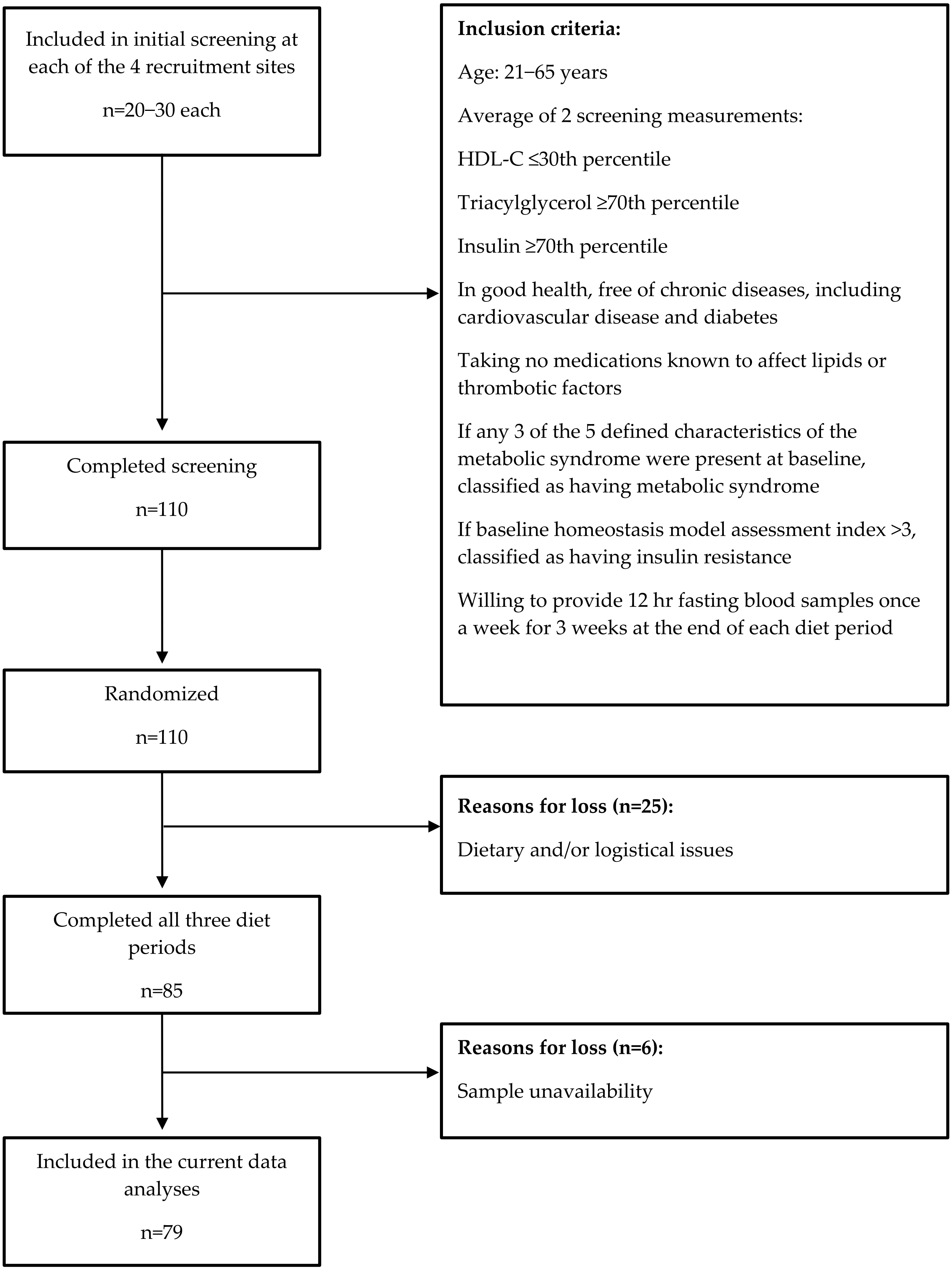

Included in initial screening at each of the 4 recruitment sites
n=20–30 each
Inclusion criteria:
Age: 21–65 years
Average of 2 screening measurements:
HDL-C ≤30th percentile
Triacylglycerol ≥70th percentile
Insulin ≥70th percentile
In good health, free of chronic diseases, including cardiovascular disease and diabetes
Taking no medications known to affect lipids or thrombotic factors
If any 3 of the 5 defined characteristics of the metabolic syndrome were present at baseline, classified as having metabolic syndrome
If baseline homeostasis model assessment index >3, classified as having insulin resistance
Willing to provide 12 hr fasting blood samples once a week for 3 weeks at the end of each diet period
Completed screening
n=110
Randomized
n=110
Reasons for loss (n=25):
Dietary and/or logistical issues
Completed all three diet periods
n=85
Reasons for loss (n=6):
Sample unavailability
Included in the current data analyses
n=79

Supplemental Figure S2. Heatmap of intraindividual changes in response to diet interventions for the most significantly changed Lp(a) lipids in men and women in DELTA 1.

(a) Men

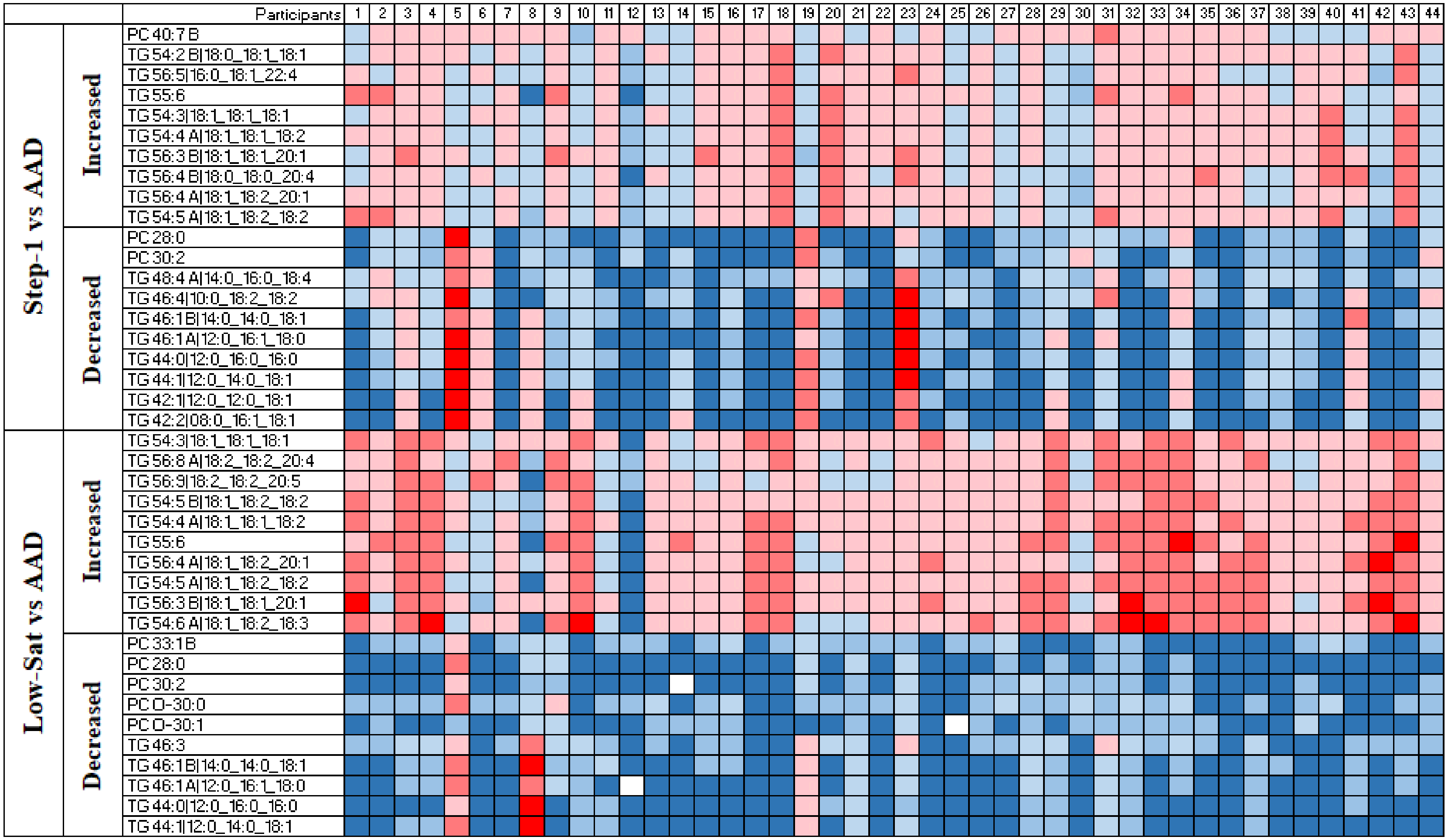

(b) Women

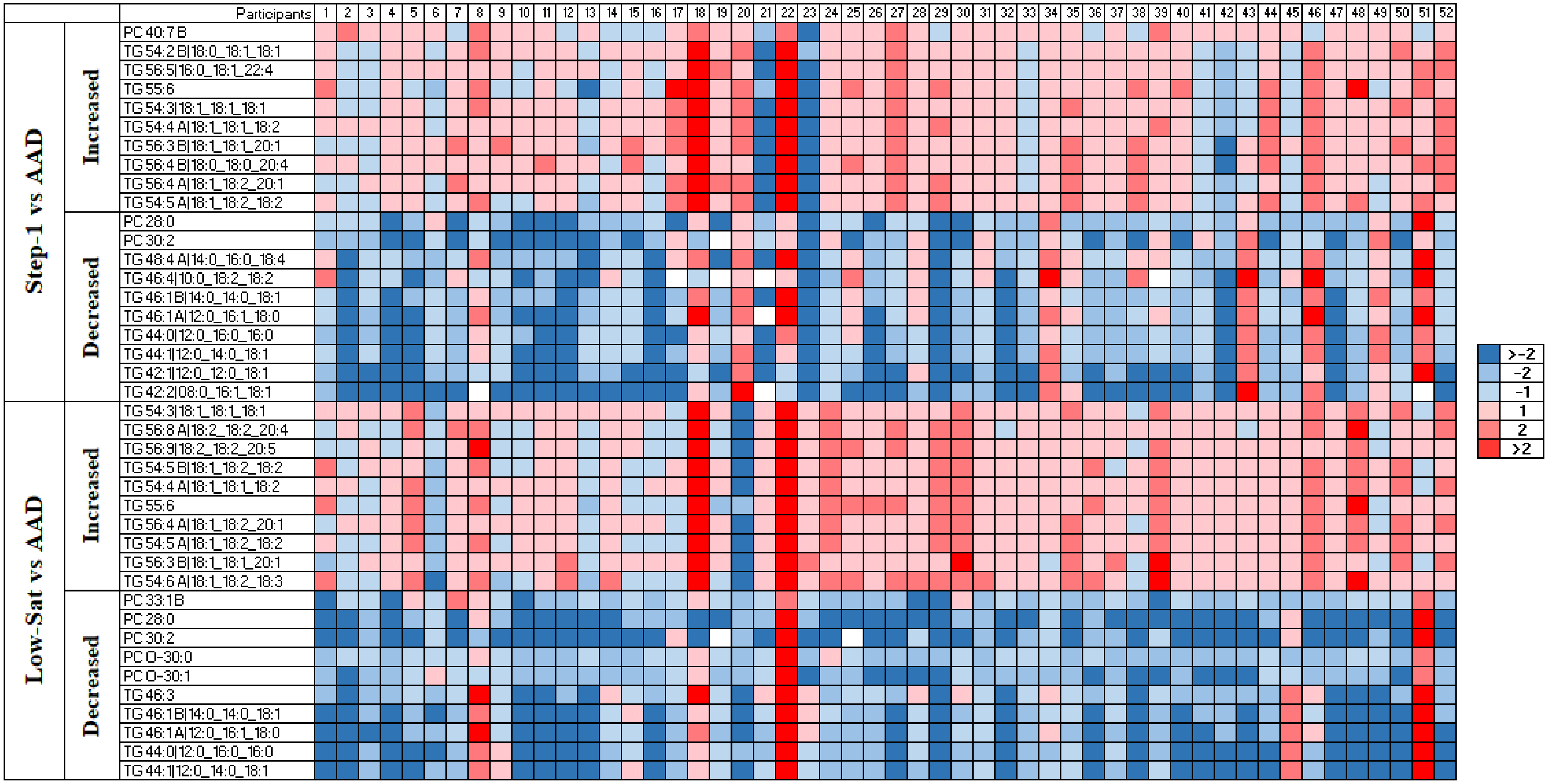

Intraindividual changes are shown as log2 transformed fold changes (log_2FC) for the most significantly changed Lp(a)-associated lipid species in (a) men (n=44) and (b) women (n=52) in DELTA 1. False discovery rate adjusted *p*-values are <0.05 for all. Fold changes were estimated as described in the methods section and log_2 transformed to demonstrate the direction of effect. Red squares indicate increase, blue squares indicate decrease, and blank squares indicate missing data or values that are non-detectable. Scale bar shows the color intensity to corresponding log_2 transformed fold changes. Annotations A and B represent isomers. Abbreviations: AAD, average American diet; Step-1, Step-1 diet; Low-Sat, low-saturated fat diet.

Supplemental Figure S3. Heatmap of intraindividual changes in response to diet interventions for the most significantly changed Lp(a) lipids in men and women in DELTA 2

(a) Men

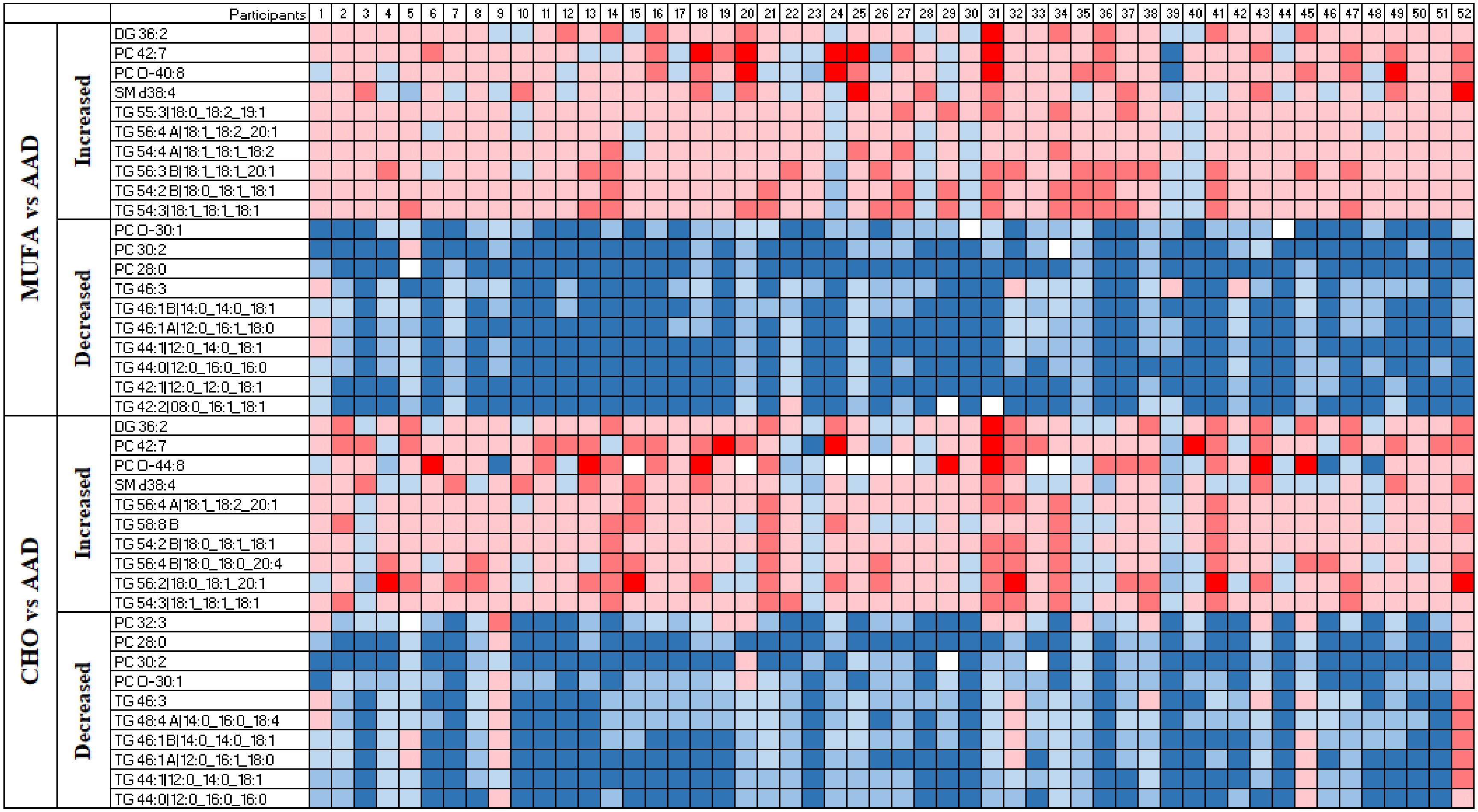

(b) Women

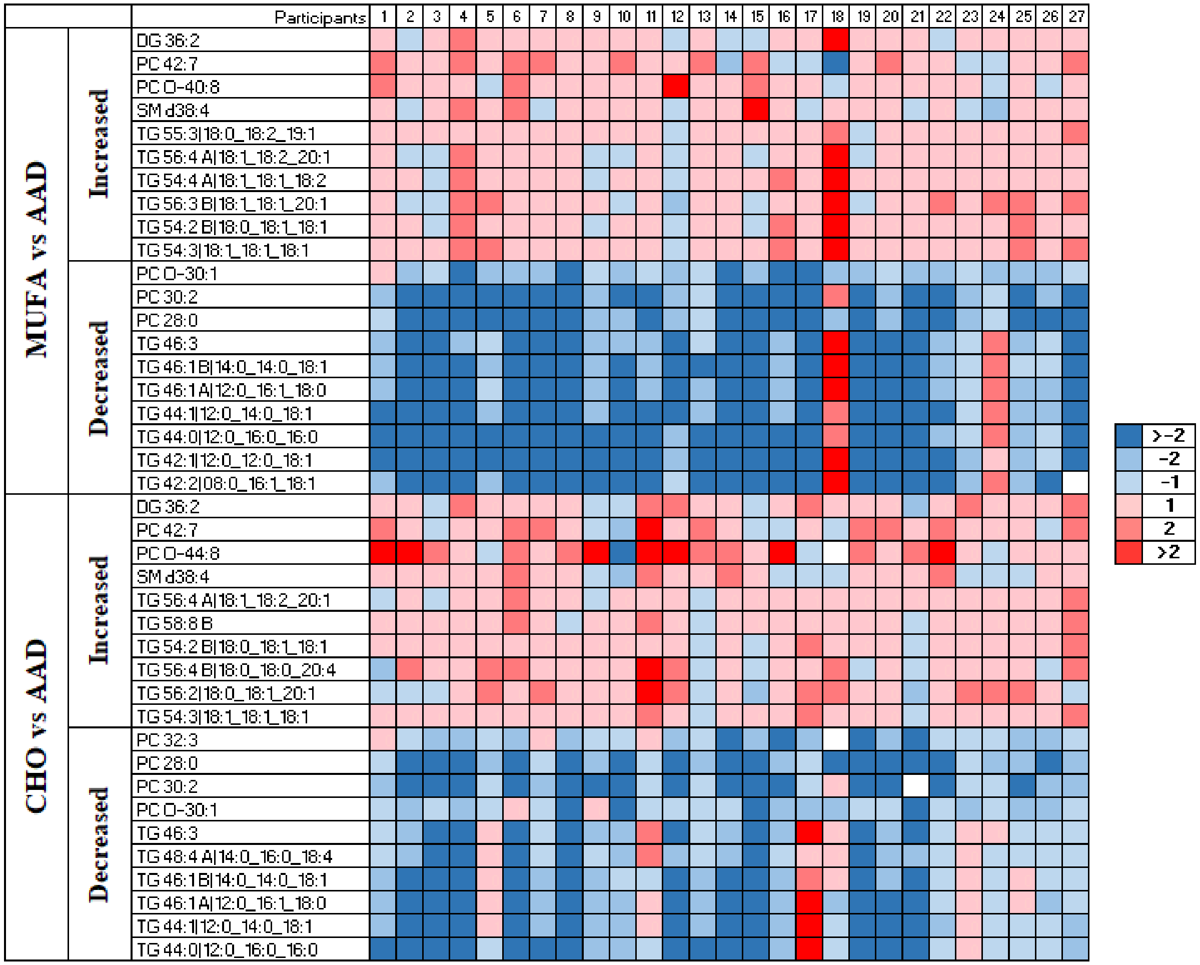

Intraindividual changes are shown as log_2 transformed fold changes (log_2FC) for the most significantly changed Lp(a)-associated lipid species in (a) men (n=52) and (b) women (n=27) in DELTA 2. False discovery rate adjusted *p*-values <0.05 for all. Fold changes were estimated as described in the methods section and log_2 transformed to demonstrate the direction of effect. Red squares indicate increase, blue squares indicate decrease, and blank squares indicate missing data or values

that are non-detectable. Scale bar shows the color intensity to corresponding log_2 transformed fold changes. Annotations A and B represent isomers.

Abbreviations: AAD, average American diet; MUFA, monounsaturated fatty acid diet; CHO, carbohydrate diet.

Supplemental Figure S4. 3D scores plots from principal component analysis in DELTA 1 and 2

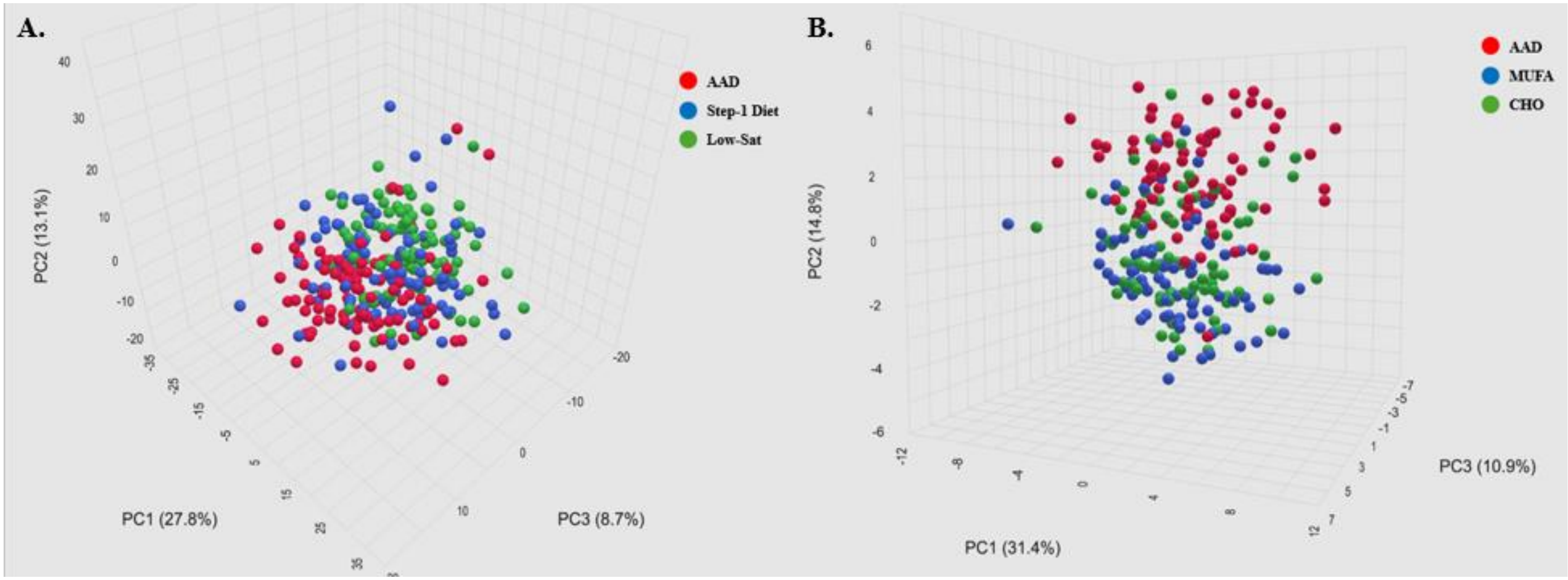

Unsupervised principal component analysis (PCA) 3D scores plots of Lp(a) lipidomic profiles in: (a) DELTA 1 and (b) DELTA 2. In DELTA 1, each point represents an individual sample, colored by diet group: red (AAD), blue (Step-1 diet), and green (Low-Sat). The first three components (PC1, PC2, and PC3) represent 49.6% of total variance. In DELTA 2, each point represents an individual sample, colored by diet group: red (AAD), blue (MUFA), and green (CHO). The first three components (PC1, PC2, and PC3) represent 57.1% of total variance. Abbreviations: AAD, average American diet; Step-1, Step-1 diet; Low-Sat, low-saturated fat diet, MUFA, monounsaturated fatty acid diet; CHO, carbohydrate diet.
